# New compounds from heterocyclic amines scaffold with multitarget inhibitory activity on Aβ aggregation, AChE, and BACE1 in the Alzheimer disease

**DOI:** 10.1371/journal.pone.0269129

**Published:** 2022-06-03

**Authors:** Iohanan Daniel García Marín, Raúl Horacio Camarillo López, Oscar Aurelio Martínez, Itzia Irene Padilla-Martínez, José Correa-Basurto, Martha Cecilia Rosales-Hernández

**Affiliations:** 1 Laboratorio de Biofísica y Biocatálisis, Escuela Superior de Medicina, Instituto Politécnico Nacional, Ciudad de México, México; 2 Dirección Académica de Ingenierías Civil, Mecánica, Química, Ambiental y Sustentabilidad, Universidad Tecnológica de México, Campus Sur, Ciudad de México, México; 3 Laboratorio de Investigación en Química Orgánica y Supramolecular, Unidad Profesional Interdisciplinaria de Biotecnología del Instituto Politécnico Nacional, Ciudad de México, México; 4 Laboratorio de Diseño y Desarrollo de Nuevos Fármacos e Innovación Biotécnológica (Laboratory for the Design and Development of New Drugs and Biotechnological Innovation), Escuela Superior de Medicina, Instituto Politécnico Nacional, Ciudad de México, México; Torrey Pines Institute for Molecular Studies, UNITED STATES

## Abstract

The preset neurodegenerations in Alzheimer disease (AD) are due to several mechanisms such as amyloidogenic proteolysis, neuroinflammation, mitochondrial dysfunction, neurofibrillary tangles, cholinergic dysfunction, among others. The aim of this work was to develop multitarget molecules for the treatment of AD. Therefore, a family of 64 molecules was designed based on ligand structure pharmacophores able to inhibit the activity of beta secretase (BACE1) and acetylcholinesterase (AChE) as well as to avoid amyloid beta (Aβ_1–42_) oligomerization. The backbone of designed molecules consisted of a trisubstituted aromatic ring, one of the substituents was a heterocyclic amine (piperidine, morpholine, pyrrolidine or *N*-methyl pyrrolidine) separated from the aromatic system by three carbon atoms. The set of compounds was screened *in silico* employing molecular docking calculations and chemoinformatic analyses. Based on Gibbs free energy of binding, binding mode and *in silico* predicted toxicity results, three of the best candidates were selected, synthesized, and evaluated *in vitro*; **F3S4-m**, **F2S4-m,** and **F2S4-p**. All three compounds prevented Aβ_1–42_ aggregation (F3S4-m in 30.5%, F2S4-p in 42.1%, and F2S4-m in 60.9%). Additionally, inhibitory activity against AChE (ki 0.40 μM and 0.19 μM) and BACE1 (IC_50_ 15.97 μM and 8.38 μM) was also observed for compounds **F2S4-m** and **F3S4-m**, respectively. Despite the BACE IC_50_ results demonstrated that all compounds are very less potent respect to peptidomimetic inhibitor (PI-IV IC_50_ 3.20 nM), we can still say that **F3S4-m** is capable to inhibit AChE and BACE1.

## 1. Introduction

Alzheimer’s disease (AD) is a cognitive disorder produced by the decrease of neurons and synaptic connectivity [1]. Over the years, and with the help of numerous investigations, the multifactorial nature of AD has been confirmed and many theories to understand its development have been raised [2]. While some theories focus on how gut-microbiota and oxidative stress play a key role in neurological conditions [3, 4], others study receptor proteins involved in the pathogenesis of AD [5], such as the amyloidogenic pathway, the hyperphosphorylation of Tau protein pathway, and some other theories attempt to get an understanding of the molecular mechanism of the cholinergic system which is involved in the failure of cognition [6–10].

The amyloid hypothesis involves the presence of extracellular plaques formed by amyloid beta peptide (Aβ) of lengths between 39 to 43 amino acid (aa) residues, with molecular weight 4–6 KDa. However, Aβ of 42 aa residues (Aβ_1–42_) represents the most amyloidogenic and toxic form [11]. The Aβ is the natural product of the metabolism of the amyloid precursor protein (APP) which is present in neurons and can be processed by the action of several proteases by two pathways. The most common route is the non-amyloidogenic pathway, where the APP is processed by the catalysis of α-secretase and γ-secretase. However, in the AD most of the APP is processed by the amyloidogenic pathway where APP is hydrolyzed by β-secretase-1 (BACE1) and γ-secretase. The hydrolysis of APP by BACE1 allows the Aβ peptide formation [12]. For this reason, several of the current therapies are focused on the inhibition of BACE1 to decrease Aβ production [13]. Once Aβ_1–42_ is released, it undergoes conformational changes from α-helix to β-sheet conformation, where the limiting event is the formation of an electrostatic interaction between the lateral chains of Asp23 or Ala42 and Lys28 acquiring a β-turn or S-shape which allow intermonomer interaction forming oligomers and fibrils of Aβ_1–42_ [14, 15]. The Aβ_1–42_ oligomers and fibrils deposited in the brain induce cytotoxicity at pre-frontal cortex, the hippocampus, the amygdala, and the Basal nucleus of Meynert [16]. Some of these brain areas are related to memory where an important cholinergic innervation is found acting principally the Acetylcholine (ACh) neurotransmitter hydrolyzed by acetylcholinesterase enzyme (AChE) [17]. Then, several AChE inhibitors have been approved by the Food and Drug Administration for AD treatment since AChE is responsible for degrading the ACh [18–20].

Considering that AChE and BACE1 are important targets for the treatment of AD, the catalytic sites of both enzymes had been fully elucidated [21, 22]. The main active site of AChE is the catalytic triad (His447, Glu334 and Ser203), and behind this, there is a secondary site or peripheral anion site (PAS) containing Tyr337 [23–25]. The catalytic site of BACE1 is formed by Asp32, Asp228 and Glu125 that contribute to the anionic charges observed on the interaction surface of the enzyme. Amino acids such as Tyr68 and Tyr71 are also found on BACE1, giving aromatic characteristics to the catalytic site to establish π-π interactions with aromatic rings [26, 27].

It has been shown that the majority of Aβ1–42 oligomerization inhibitors are those containing a tertiary amine and an aromatic ring [28]. Additionally, it has been suggested that the vast majority of BACE1 inhibitors establish tight hydrogen bonding network with aspartic residues (Asp32 and Asp228) located in the catalytic cleft of the enzyme and are also capable of forming other hydrogen bonds and π-π interactions with the aromatic residues also located at the catalytic site [26]. Having in mind the type of interactions required to inhibit BACE1 and Aβ_1–42_ oligomerization and considering that glutamic and aspartic residues present on the active sites of BACE1 and AChE which are negatively charged, a family of 64 new molecules were designed with a tertiary amine on its structure which is capable to protonate at physiological pH and stablish electrostatic interactions with the negative catalytic site of the enzymes. Other chemical moieties were also incorporated in the structure of ligands aiming to establish hydrogen bonding, hydrophobic and π-π interactions. This family of compounds contains a heterocyclic amine such as piperidine, morpholine, pyrrolidine or N-methyl pyrrolidine linked three atoms away from a trisubstituted aromatic ring with different functional groups such as secondary or primary amines, aliphatic moieties, ester, or alcohol groups. In addition, the molecular weight of designed molecules was less than 300 g/mol. All designed molecules were screened *in silico* by molecular docking studies towards the three selected molecular targets (AChE, BACE1 and Aβ_1–42_) based on the Gibbs free energy results (ΔG) and in the binding mode, as well as in the toxicity results, and the Blood Brain Barrier (BBB) permeability measured under chemoinformatic studies, three of the best compounds named **F2S4-m**, **F2S4-p** and **F3S4-m** were selected for synthesis and evaluated *in vitro*. The results showed that **F3S4-m** was the best compound to inhibit both BACE1 and AChE activities and was able to avoid the aggregation of Aβ_1–42_, despite not having the best performance to inhibit Aβ_1–42_ aggregation as the other two compounds.

## 2. Materials and methods

### 2.1. *In silico* studies

#### 2.1.1. Target selections

The 3D structures of the proteins were retrieved from Protein Data Bank (PDB: http://www.rcsb.org/pdb/home/home.do) for docking studies; BACE1 (PDB ID: 2QP8), AChE (PDB ID: 4PQE) and Aβ_1–42_ in α-helix with PDB ID: IZ0Q, Aβ_1–42_ in β-Sheet with PDB ID: 2BEG and the Random Coil (RC) conformation reported by our research group [29]. These proteins were prepared by adding polar hydrogens and Kollman charges, meanwhile, water molecules and other ligands were removed to be then prepared for docking studies employing the AutoDock 4.4 software (AutoDock. http://autodock.scripps.edu/2019).

#### 2.1.2 Ligands design

**Fig 1:**
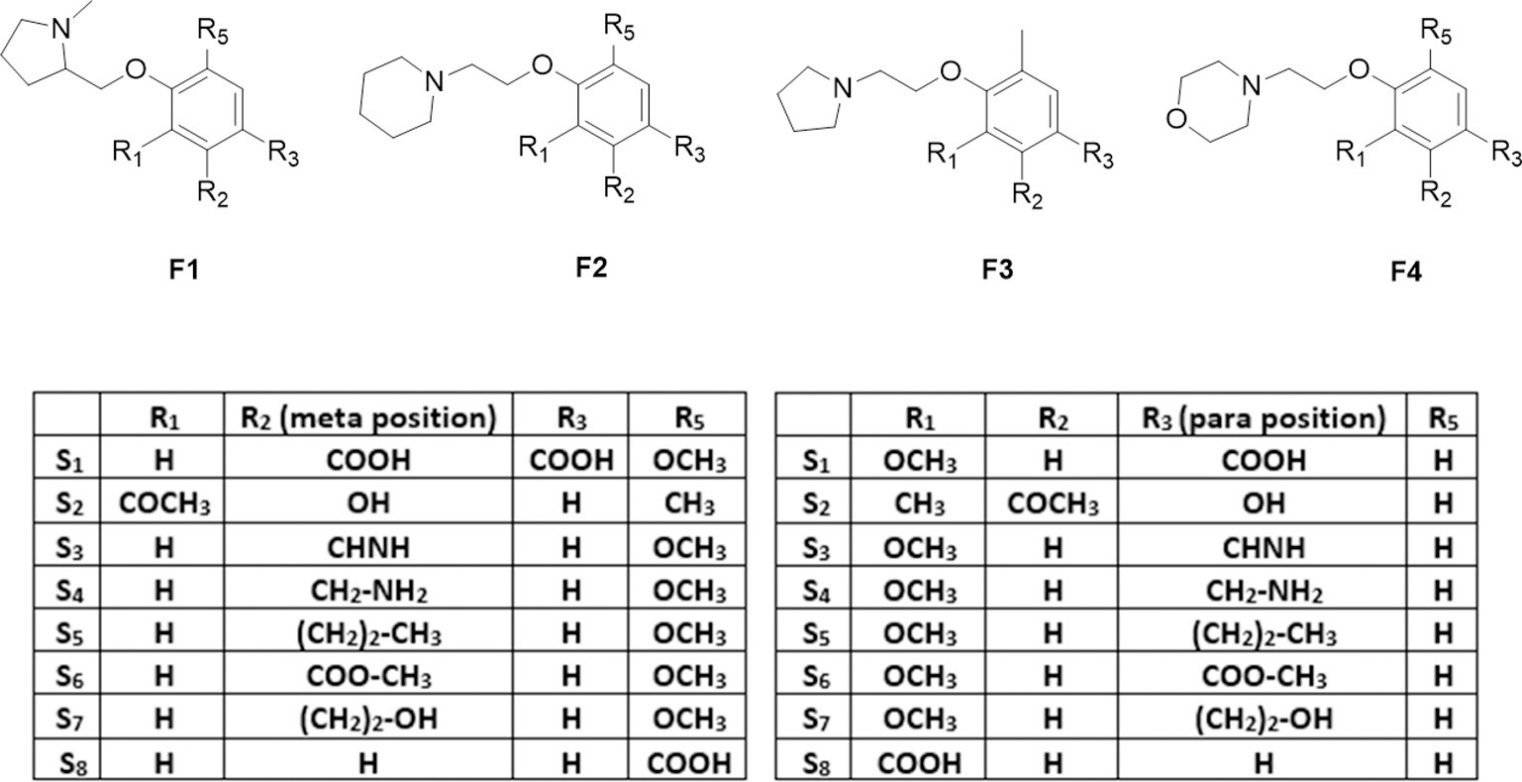
Chemical structures of ligands designed with heterocyclic amines; methyl pyrrolidine (F1), piperidine (F2), pyrrolidine (F3) and morpholine (F4) distanced 3 atoms away from the aromatic backbone. Substitution of the aromatic system with benzoic acid (S1 and S8), hydroxybenzoic acid (S2), phenylmetanimine (S3), phenylmethanamine (S4), butylbenzene (S5), methyl benzoate (S6) and phenyl ethanol (S7) in a -m or -p position related to the heterocyclic amine.

Ligands were designed with pharmacophores capable of generating either a positive electronic density or a positive charge able to interact with the anionic region of the catalytic target sites. This strategy was expected to induce not only inhibition of BACE1 and AChE but also to avoid Aβ_1–42_ oligomerization. The chemical moieties groups employed in ligands contain a tertiary amine such as, N-methyl pyrrolidine (**F1**), pyrrolidine (**F3**), piperidine (**F2**) and morpholine (**F4**) able to protonate at physiological pH and attached three atoms away from an aromatic ring backbone. In addition, the aromatic could allow the establishment of π-π interactions, also the aromatic ring was substituted with secondary or primary amines, aliphatic moieties, ester, or alcohol groups; these substituents were attached in a 1,3-(-m) or 1,4 (-p) position regarding the amine. These substitutions led to 64 compounds grouped in four families of molecules named as **F**_**x**_**S**_**y**_**-m/p** where **F**_**x**_ is the type of tertiary amine employed, **S**_**y**_ is the substituent attached to the aromatic ring and -**m/p** is the substituent in meta or para position relative to the tertiary amine (Fig 1). For the in-silico studies a positive control for each protein was employed; peptidomimetic inhibitor IV (**IP-IV**) [30], galantamine and curcumin, for BACE1, AChE and Aβ, respectively.

#### 2.1.3 Docking analyses

All ligands were drawn in 2D using ChemBioDraw Ultra 12.0 (ChemBioDraw Ultra. http://chembiodraw-ultra.software.informer.com/, 2019), and their geometry was pre-optimized using Hyperchem (HyperChem, HYPERCUBE, INC. http://www.hyper.com/, 2019) at the molecular mechanic level. AutoDock tools 1.5.6 (AutoDock. http://autodock.scripps.edu/, 2019) were used to prepare docking studies with a grid box of 60 Å^3^ on the catalytic site for each protein. The Glu22 and Asp23 are exposed in all the three different conformations of Aβ_1–42_; whereas for BACE1, Asp32 and Asp228 are key residues in the catalytic site [26]. In the case of AChE, a midpoint between the primary catalytic site comprised by His447, Glu334, Ser203 and a secondary cavity containing Tyr337 was considered for the docking studies. Lamarckian genetic algorithm was employed to perform the search with an initial population of 100 random individuals and 1x10^7^ iterations were executed with AutoDock tools software, the remaining parameters were kept at their default values (AutoDock. http://autodock.scripps.edu/, 2019). The free energy values results were analyzed with AutoDockTools (AutoDock. http://autodock.scripps.edu/, 2019) software, meanwhile the biding poses were analyzed using PyMOL software (PyMOL, A Molecular Visualization System on an Open-Source Foundation, Maintained and Distributed by Schrodinger, 2019 (http://www.pymol.org). To validate the docking procedure, each enzyme was docked with their corresponding inhibitors: Galantamine for AChE, curcumin for Aβ_1–42_ and PI-IV for BACE1 [30].

#### 2.1.4 Physicochemical properties evaluated *in silico*

The OSIRIS Property Explorer (Osiris property explorer server “http://www.organic-chemistry.org) and Molinspiration Cheminformatics 2019 (“http://www.molinspiration.com”) servers were employed for chemoinformatic studies to predict the physicochemical properties of ligands according to Lipinski´s rule. Those ligands with characteristics favoring the absorption and distribution after oral administration were taken into consideration [31].

#### 2.1.5 Blood-brain-barrier permeability prediction

To predict nervous system permeability, the Blood Brain Barrier Permeability Predictor server (http://www.cbligand.org/BBB/index.php) was employed using an AdaBoost algorithm, in combination with the MACSS fingerprint. Additionally we employed a Light BBB (http://ssbio.cau.ac.kr/software/bbb) server based on previously known structures through a machine learning algorithm and SwissADME (http://www.swissadme.ch/) server based on the boiled egg experiment for brain permeability prediction [32–34]. For each server, the protonation state at pH = 7.4 of each structure was predicted and then the SMILEs code was used.

### 2.2 Chemical synthesis

All chemical starting materials purchased from Sigma-Aldrich were directly used without further purification. All solvents were distilled prior to use and acetone was dried following standard procedures.

Mass spectra were recorded by staff of Centro de Nanociencias y Micro y Nanotecnologías at the National Polytechnic Institute (https://www.ipn.mx/nanocentro/). High resolution mass spectra were recorded on a micOTOF-Q mass spectrometer. Thin layer chromatography (TLC) was performed using commercially available pre-coated plates (TLC Silica gel 60 F₂₅₄). Column chromatography was conducted using Merck Silica gel 60 (0.063–0.100 mm). Preparative thin layer chromatography (prep-TLC) was performed using commercially available pre-coated plates (TLC Aluminum oxide 60 F₂₅₄, neutral).

The synthesized compounds were characterized by ^1^H and ^13^C NMR spectroscopy. All spectra were recorded on a Varian 300 MHz spectrometer. Samples of compounds were dissolved in the indicated deuterated solvent; chemical shifts (δ) were reported in ppm downfield to tetramethyl silane. Coupling constants (J) are reported in Hertz and rounded to 0.1 Hz. Splitting patterns are abbreviated as follows: singlet (s), doublet (d), triplet (t), quintet (q), multiplet (m), broad (br) or a combination of these.

**Scheme 1:**
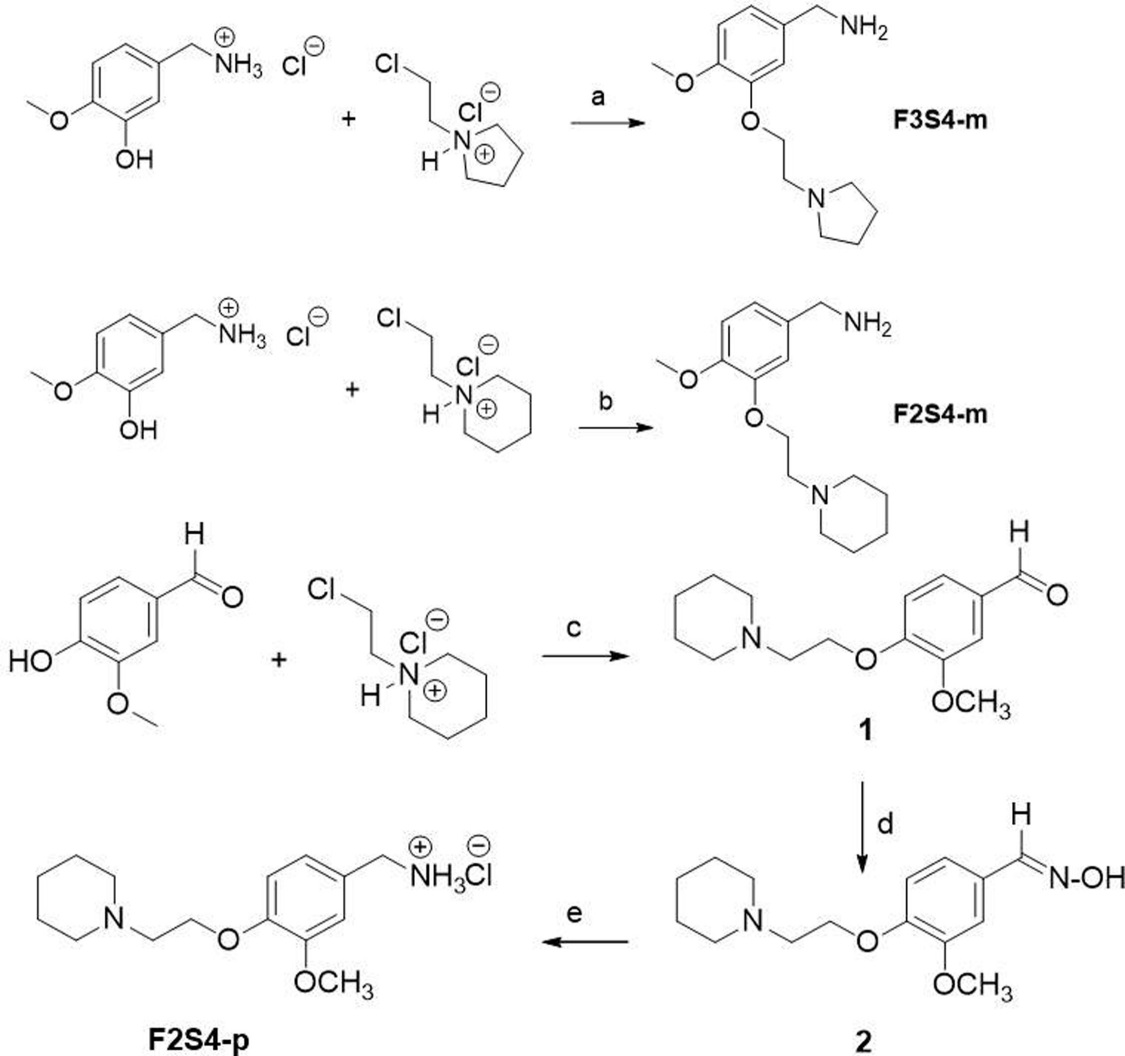
General reaction for F3S4-m, F2S4-m, and F2S4-p formation. Reagents (K_2_CO_3_, dry acetone) and conditions: (a) reflux 48 h, (b) reflux 12 h, (c) reflux 8 h; (d) NaHCO_3_, EtOH, reflux 4 h; (e) Pd/C 10%, 70 psi, absolute EtOH, 4 h.

Based on the *in-silico* results which include docking and the best physicochemical properties according to the Lipinsky rules, compounds **F3S4-m**, **F2S4-m** and **F2S4-p** were the most promissory to be selected for synthesis following the methodology shown in Scheme 1. The methenamine compounds **F3S4-m** and **F2S4-m** were obtained employing the Williamson ether synthesis, which involves the reaction of an alkoxide with a primary alkyl halide via a nucleophilic substitution reaction. The synthesis of compound **F2S4-p** was performed following a different approach to obtain the amine group in *para* position relative to the alkoxy group. Initially, the Williamson ether synthesis was performed employing the 4-hydroxy-3-methoxybenzaldehyde (Vanillin), as the starting material. The next step required the formation of the appropriate oxime which ultimately was further reduced to the desired amine employing a catalytic hydrogenation procedure.

#### 2.2.1. Synthesis of (4-methoxy-3-(2-(pyrrolidin-1-yl) ethoxy) phenyl) methanamine (F3S4-m)

Finely grounded K_2_CO_3_ (2914 mg, 21 mmol) was added to a mixture of 3-hydroxy-4-methoxybenzylamine hydrochloride (2000 mg, 10.5 mmol) and 1-(2-chloroethyl) pyrrolidine hydrochloride (1790 mg, 10.5 mmol) dissolved in dry acetone (30 mL). The progress of reaction was monitored by TLC until completion. The mixture reacted under refluxing conditions for 48 h protected from moisture employing a calcium chloride trap. After cooling the reaction to room temperature, the K_2_CO_3_ was removed by vacuum-filtration and the solvent was removed under reduced pressure. The resulting viscous residue was then purified by alumina gel column chromatography using MetOH as eluent, obtaining a light-brown sticky residue which then was subjected to solvent removal in a Schlenk line under vacuum overnight, to afford **F3S4-m** (1324 mg) as a light-brown powder, solubility MetOH, EtOH, Acetone (75.6% yield, 96% purity). IR (cm^-1^): 2929, 2957, 1575, 1136, 1442, 1260. ^1^H NMR (300 MHz, CDCl_3_) δ 7.20 (s, 1H, H-2), 6.94 (dd, ^3^J = 8.4, 1.3, 1H, H-6), 6.81 (d, ^3^J = 8.3, 1H, H-5), 4.59 (a, 3H, NH_3_), 4.36 (t, ^3^J = 5.7, 2H, H-7), 3.92 (s, 2H, H-11), 3.80 (s, 3H, OCH_3_), 3.30 (t, 2H, H-8), 3.08 (br, 4H, H-9), 1.97 (br, 4H, H-10). ^13^C NMR (75.45 MHz, CDCl_3_) δ 148.5, 148.0, 120.2 112.9, 111.6, 67.2, 55.9, 54.7, 54.5, 45.4, 23.4. HR-MS m/z for C1_4_H_22_N_2_O_2_, calculated 250.1681, found; 251.1622 [M+H]^+^. Decomposition was observed after 250°C. compound (1324 mg, yield 75%).

#### 2.2.2. Synthesis of (4-methoxy-3-(2-(piperidin-1-yl) ethoxy) phenyl) methanamine (F2S4-m)

For the syntheses of **F3S4-m** the following quantities: K_2_CO_3_ (435 mg, 3.15 mmol), 3-hydroxy-4-methoxybenzylamine hydrochloride (300 mg, 1.60 mmol), 1-(2-chloroethyl) piperidine hydrochloride (316 mg, 1.70 mmol), dry acetone (40 mL). The mixture was reacted for 12 h under nitrogen atmosphere. The obtained yellow viscous residue (565 mg) was purified by preparative thin layer chromatography (TLC). Four aluminium oxide plates (20 X 20 cm) were employed to purify 200 mg of crude mixture. The plates were eluted with EtOAc/MetOH (8:2). The layer with an R_f_ around 0.2 (observed under UV-light) was scrapped out from the plates, the aluminium oxide was then stirred with methanol for at least 30 min and then vacuum-aid filtrated. The organic solvent was removed under reduced pressure to yield a light-yellow sticky slurry which then was subjected to solvent removal in a Schlenk line under vacuum overnight to finally obtaining compound **F2S4-m** (109 mg, 0.412 mmol, 54.5% yield, 95% purity). Solubility; MetOH, EtOH, Acetone, CHCl_3_, EtOAc, H_2_O. IR (cm^-1^): 2930.6, 2852–2832, 1513, 1441, 1259, 1136, 806.6. ^1^H NMR (300 MHz, CDCl_3_) δ 6.88 (s, 1H, H-2), 6.83 (br, 2H, H-5, H-6), 4.15 (t, ^3^J = 6.2, 1H, H7), 3.84 (s, 3H, OMe), 3.78 (s, 2H, PhCH_2_), 2.81 (t, ^3^J = 6.3, 2H, H-8), 2.50 (br, 4H, H-9), 1.60 (q, ^3^J = 5.5, 4H, H-10), 1.44 (q, ^3^J = 5.9, 2H, H-11). ^13^C NMR (75.45 MHz, CDCl_3_) δ 148.4 and 148.3 (C-3 y C-4), 136.2 (C-1), 119.4 (C-6), 112.3 and 111.7 (C-2 y C-5), 66.4 (C-7), 57.9 (OMe), 56.2 (PhCH_2_), 55.1 (C-9), 46.3 (C-8), 26.0 (C-10), 24.3 (C-11). MS (ESI): m/z calculated for C_15_H_25_ClN_2_O_2_: 300.1605 (hydrochloride), C_15_H_24_N_2_O_2_: 264.1838 (free amine); found: 265.1911 g/mol [M+H]^+^.

#### 2.2.3. Synthesis of (3-methoxy-4-(2-(piperidin-1-yl) ethoxy) benzaldehyde (1)

Synthesized as described for **F3S4-m** using the following quantities: K_2_CO_3_ (276 mg, 20.0 mmol), 4-hydroxy-3-methoxy benzaldehyde (152 mg, 10.0 mmol) and 1-(2-chloroethyl) piperidine hydrochloride (276 mg, 15.0 mmol), dry acetone (200 mL). The mixture reacted for 8 h. The desired product was obtained as an oily, brown-coloured residue which then remained in a Schlenk line under vacuum overnight to remove solvent traces and finally obtaining compound **1** (65% yield, 92.5% purity). No further purification was required. Solubility; MetOH, EtOH, CHCl_3_, CH_2_Cl_2_. ^1^H NMR (300 MHz, CDCl^3^) δ 9.79 (s.1H, CHO), 7.37 (d.1H, ^3^J = 3 Hz, H-5), 7.35 (dd, 1H, ^3^J = 3, 9 Hz, H-6), 6.96 (d, 1H, ^3^J = 9 Hz, H-2), 4.20 (t, 2H, ^3^J = 6 Hz, H-7), 3.87 (s, 3H, OMe), 2.82 (t, 2H, ^3^J = 6 Hz, H-8), 2.5 (t, 4H, ^3^J = 6 Hz, H-9), 1.58 (q, 4H, ^3^J = 6 Hz, H-10), 1.41 (q, 2H, ^3^J = 3 Hz, H-11). MS (ESI): m/z calculated for C_15_H_21_N O_3_: 263.1521, found 264.1598 [M+H]^+^.

#### 2.2.4. Synthesis of (*E*)-3-methoxy-4-(2-(piperidin-1-yl) ethoxy) benzaldehyde oxime (2)

Finely grounded NaHCO_3_ (143 mg, 17.0 mmol) was added to a mixture of 3-methoxy-4-(2-(piperidin-1-yl) ethoxy) benzaldehyde (278 mg, 10.0 mmol) and hydroxylamine hydrochloride (111 mg, 16.0 mmol) dissolved in EtOH (10 mL). The mixture reacted under refluxing conditions for 4 h. The progress of reaction was monitored by TLC, once completed, the mixture was allowed to cool down to room temperature followed by addition of distilled water (100 mL). The resulting white precipitate was filtered with the aid of vacuum-filtration. The desired product was then dried in a Schlenk line under vacuum overnight to obtain a white solid (60% yield, 97% purity). MetOH, EtOH, CHCl_3_, CH_2_Cl_2_. mp 119.6–120.8°C. ^1^H NMR (300 MHz, CDCl3) δ 10.77 (s.1H, NOH), 8.02 (s, 1H CH = NOH), 7.00 (dd.1H, ^3^J = 3, 9 Hz, H-6), 6.95 (d, 1H, ^3^J = 3 Hz, H-2), 6.87 (d, 1H, ^3^J = 9 Hz, H-5), 4.29 (t, 1H, ^3^J = 6 Hz, H-7), 3.78 (s, 3H, OMe), 2.85 (t, 2H, ^3^J = 6 Hz, H-8), 2.58 (t, 4H, ^3^J = 6 Hz, H-9), 1.63 (q, 4H, ^3^J = 6 Hz, H-10), 1.43 (c, 2H, ^3^J = 3 Hz, H-11). MS (ESI): m/z calculated for C_15_H_22_N_2_ O_3_: 278.1630, found 279.1709 [M+H]^+^.

#### 2.2.5. Synthesis of (3-methoxy-4-(2-(piperidin-1-yl) ethoxy) phenyl) methanamine (F2S4-p)

To a solution of (E)-3-methoxy-4-(2-(piperidin-1-yl) ethoxy) benzaldehyde oxime (1.8 g, 6.5 mmol) dissolved in absolute EtOH (162.5 mL) was added 325 mg of Pd/C (10%). The mixture was allowed to react under catalytic hydrogenation (70 psi, 4h). The chemical reaction was monitored by TLC, once completed; the reducing agent was removed by filtration. The solvent was removed under reduced pressure to afford the desired product as a pale-yellow powder (94% yield, 95% purity). **S**solubility; MetOH, EtOH, water. mp 230.2–231.5°C. IR (cm^-1^): 3000–2800 2640. ^1^H NMR (300 MHz, CD_3_OD) δ 7.20 (d, 1H, ^3^J = 3, H-2), 7.05 (dd, 1H, ^4^J = 3, ^3^J = 6, H-5), 7.04 (dd, 1H, ^4^J = 3, ^3^J = 6, H-6), 4.97 (t, 2H, ^3^J = 6, Ar-CH_2_-**NH**_**2**_), 4.33 (s, 2H, ^3^J = 6, H-7), 4.08 (t, 2H, ^3^J = 6, Ar-**CH**_**2**_-NH_2_), 3.91 (s, 3H, OMe), 3.36 (t, 2H, ^3^J = 6, H-8), 3.18 (t, 4H, ^3^J = 6, H-9), 1.86 (q, 4H, ^3^J = 6, H-10), 1.64 (q, 2H, ^3^J = 3, H-11). ^13^C NMR (75 MHz, CD_3_OD) 151.52 (6 CH**Ar**), 149.49, 129.03, 123.00, 116.16 and 114.18, 66.81 (N-CH_2_-**CH**_**2**-_O) and 57.56 (N-**CH**_**2**_-CH_2-_O), δ = 56.81 (OCH_3_), 55.43 (CH_2_-(CH_2-_**CH**_**2**_)_2_-N), 44.39 (Ar-**CH**_**2**_-NH_2_), 25.05 (CH_2_-(**CH**_**2**-_CH_2_)_2_-N), δ 23.49 (**CH**_**2**_-(CH_2-_CH_2_)_2_-N). MS (ESI): m/z calculated for C_15_H_24_N_2_ O_2_: 264.1838, found 265.1923 [M+Na].

All spectra are shown in the supporting information as S1-S12 Figs in S1 File.

### 2.3 *In vitro* studies

#### 2.3.1 Inhibition of Aβ_1–42_ aggregation by Thioflavin T (ThT) assay

Evaluation of ligands as Aβ_1–42_ fibril formation inhibitors was performed as follows: a solution of Aβ_1–42_ (Calbiochem, Cat. No. PP69) at 0.25 μg/μL in milliQ water, was incubated with or without each one of the selected compounds (**F2S4-p**, **F2S4-m**, **F3S4-m**) at 100 μM in a quartz cell at 37°C, the mixture (300 μL) was constantly shaken over 48 h. Aliquots (150 μL) from this solution were taken at 48 h. 25 μL of ThT at 3.0 mM was added and diluted to a final volume of 600 μL with water. The increase in ThT fluorescence was measured at λ emission = 480 nm and λ excitation = 445 nm [28]. Fluorescence emission was recorded using an LS-55 Spectrofluorometer (PerkinElmer), equipped with a water jacketed cell holder for temperature control. All the experiments were performed using cells with a path-length of 0.5 cm, at 37°C.

#### 2.3.2 Inhibition of BACE 1 activity

The enzymatic activity of BACE 1 was measured using a fluorescence resonance energy transfer (FRET) kit (Thermo Scientific, MA, US). All compounds were dissolved in 50 mM sodium acetate buffer pH 4.5 with 0.3% v/v of MeOH HPLC grade. Measurements were taken 1 h later of being shaken at room temperature. The synthesized inhibitors were compared against a β-Secretase **IP-IV** (Cayman Chemichal, MI, US) as positive control [30].

The fluorescence was measured in Tecan M1000 pro multimode microplate reader (Tecan Trading, ZRH, Switzerland). After blank subtraction, activity was determined from Eq 1.


$$Inhibition\;ratio=100-\left[\frac{FI\times 100}{NCF}\right]$$ (1)

Replacing the fluorescence intensity (FI) obtained in the completed reaction with each compound and the fluorescence intensity of the negative control fluorescence (NCF) corresponding to BACE1 and substrate without inhibitor.

#### 2.3.3 Acetylcholinesterase inhibition assays

The Bonting-Featherstone method [35, 36] was used to quantify the remaining ACh in the assay. A standard curve of ACh at 0.2, 0.4, 0.8, 1.6, 3.2, 6.4 and 12.8 mM diluted in 0.1M potassium phosphate buffer pH 8 was done. Samples were incubated over 1 h at 37°C under constant shaking. After 25 μL of alkaline hydroxylamine (1:1) with NaOH 14% w/v was added. Each sample was diluted 1:4 using a solution of FeCl_3_ 78 mM in 3.8 mM HCl, thereafter each sample was centrifuged at 5000 rpm/10 min, and finally the optical density of the supernatant was measured at λ = 500 nm, using the spectrophotometer multiskan sky microplate (Thermo Fisher Scientific, CA, US). Determination of AChE catalytic activity was conducted in the presence of compounds at 25, 50 and 100 μM of each inhibitor using Galantamine as positive control. For this purpose, 0.0006 U/μl of electric eel (*Electrophorus electricus*) AChE C3389 (Merck, Darmstadt, Germany) was preincubated in the presence of compounds for 1 h under constant shaking. Subsequently, the respective ACh solution was added and re-incubated for 1 h at 37°C, giving the same treatment previously reported for the standard curve. The enzyme activity was expressed as the difference in optical density between the inhibitor assays and the respective standard curve labeled as ΔAbs. ΔAbs = (Absorbance of ACh without AChE—Absorbance of blank)- (Absorbance of ACh with AChE—Absorbance of blank). Once obtained 1/ΔAbs, and 1/[ACh], a Lineweaver Burk plot (LWP) was drawn. The LWP was performed for each inhibitor concentration and the slope was plotted in function of their increase to determine inhibition constant (Ki) values.

#### 2.3.4 Evaluation of cytotoxic effect of compounds on astrocytes, PC12, and NIH3T3 fibroblasts cultures

The cell lines of astrocytes, PC12 and NIH3T3 fibroblasts were grown in essential DMEM medium with fetal bovine serum, 10% and 1X antibiotic-antifungal (penicillin G, sodium salt and 1% streptomycin sulfate) using a 5% CO_2_ atmosphere at 37°C. For manipulation and visualization, a biosafety level 2 vertical laminar flow cabinet (NUAIRE A2 NU-543-400) and an inverted binocular microscope (MOTIC AE-20) were used, respectively. To detach the cells, a PBS-trypsin solution was used. The cells were seeded in a 96 plate well with 10x10^3^ cells in each well. After, 24 h the medium was replaced, by different treatments: medium only, medium with 0.01% of DMSO and medium with **F2S4-p**, **F2S4-m,** or **F3S4-m** at 12.5, 25, 50, 100, and 200 μM. Two independent experiments were performed with n = 8 for each treatment. For the viability test, 3-(4,5-dimethylthiazol-2-yl)-2,5-diphenyltetrazole (MTT) was employed as follows; the medium was replaced with 100 μL of a MTT solution (0.5 mg of MTT /1 ml of medium). The 96 plate well was incubated for 4 h using a 5% CO_2_ atmosphere at 37°C. After, the MTT was removed and 100 μL of DMSO was added, to solubilize the formazan salts. The plate was then read in the spectrophotometer multiskan sky microplate (Thermo Fisher Scientific, CA, US) at 550 nm. Cell viability of astrocytes cell cultures in the presence of Cadmium chloride (0.01, 0.1, 1, 10, 100 μM) as control for cytotoxicity effect is shown in the supporting information as S13 Fig in S1 File. It was found that astrocytes viability diminished up to 25% at 100 μM, at concentrations 1 μM ≤ on the other hand, cell viability remained above 80%.

#### 2.3.5. Statistical analysis

The results are presented as the mean ± SE. All analyses were performed using the statistical program Sigma Stat for Windows Version 2.03 software (SPSS Inc) and the graphs were realized using GraphPad Prism Version 5.00 software.

## 3. Results

### 3.1. The compounds with methanamine substituent in the aromatic ring have better affinity on Aβ_1–42_, AChE and BACE1 by *in silico* studies

**Fig 2:**
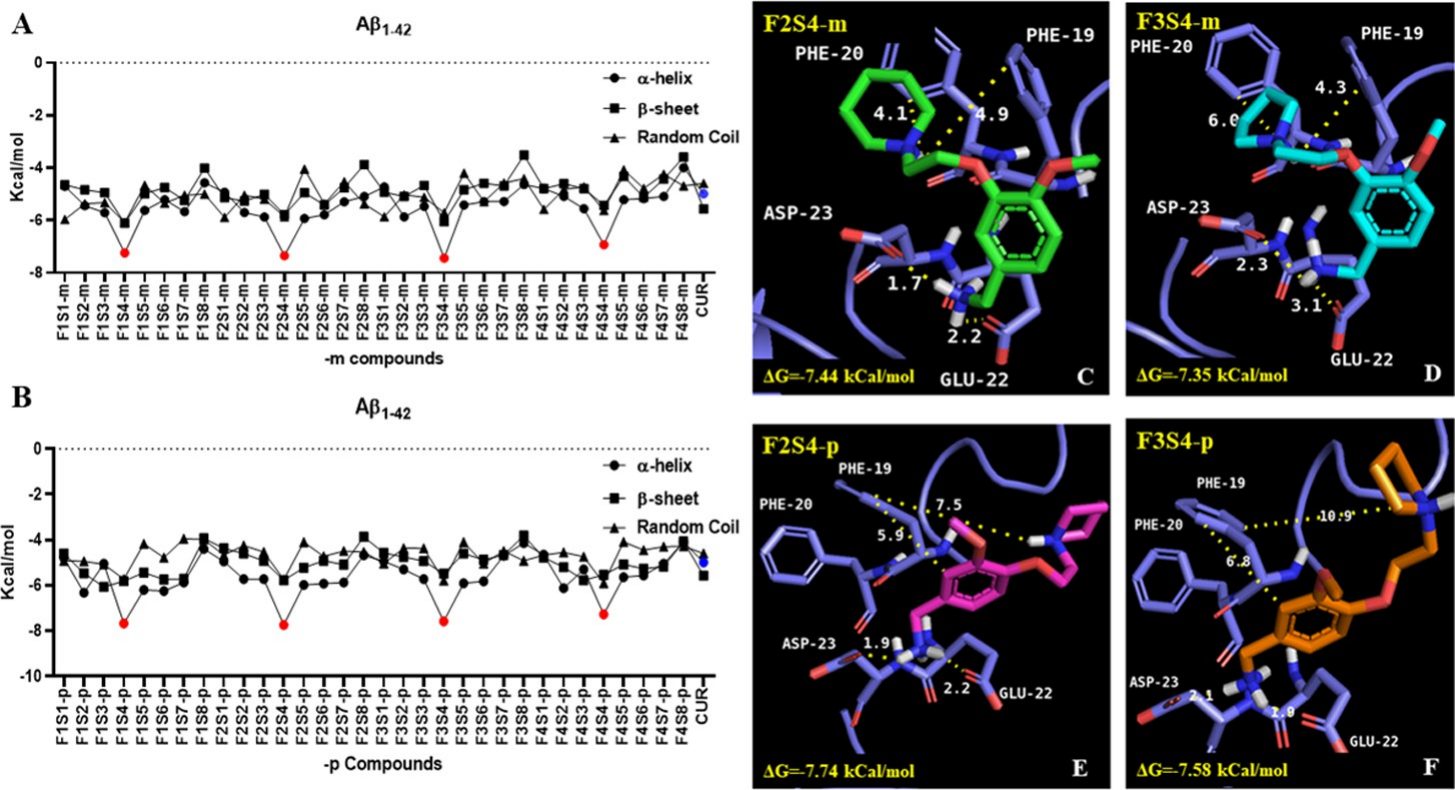
Docking results for designed compounds towards three Aβ_1–42_ monomer secondary conformations (α-helix, β-sheet, and random coil). Free energy values obtained for; A) 1,3 substituted (-meta) compounds, B) 1,4 substituted (-para) compounds. Methanamine (S4) bearing compounds in both substitution positions (red points in chart) containing piperidine (F2) and pyrrolidine (F3) were the best compounds. Binding analysis of containing methanamine compounds (S4) and Aβ_1–42_: C) F2S4-p, D) F3S4-m, E) F2S4-p, F) F3S4-p. All compounds were able to place one of amino groups in an interaction with Asp 23 and Glu 22 and interact with aromatic residues (Phe20) by π-π stackings.

According to *in silico* studies, the compounds with the best theoretical energy values were those in which the methanamine substituent is bounded to the aromatic ring backbone (**S4** group) in -*m* and -*p* position relative to the heterocyclic amine (Fig 2A and 2B). Compounds **F2S4-m** (Fig 2C), **F3S4-m** (Fig 2D), **F2S4-p** (Fig 2E) and **F3S4-p** (Fig 2F) showed a greater affinity to the α-helix conformation of Aβ_1–42_ with ΔG values as lowest as -7.35, -7.44, -7.74, -7.58 kcal /mol respectively, in comparison with Aβ_1–42_ in β-sheet and RC conformations, showing higher ΔG values (Fig 2A and 2B). In addition, compounds with **S4** group showed not only better ΔG values than curcumin, but also were more selective for Aβ_1–42_ in α-helix conformation Fig 2A and 2B. All molecules established an electrostatic interaction with Asp23 and Glu22 in the monomeric form of Aβ_1–42_ (Fig 2C–2F).

Besides the electrostatic interaction between the heterocyclic charged amine of compounds with Asp23/Glu22 in the α-helix Aβ_1–42_ conformation, other types of non-bond interaction were observed. All the nonbond interactions with the residues are shown in the S1 Table in S1 File. For example, compounds containing carboxylic acid, as observed in the in the **S1** and **S8** series, exhibited H-bond interactions with Lys28 and electrostatic interaction with Lys26. Compounds with aliphatic chains as in the **S5** series showed H-bond interactions, electrostatic interactions with Glu22 and Asp23 and more specifically with His14 for **F4S5-m**.

Compounds bearing the hydroxybenzoic acid, as in the **S2** series, established H-bond interactions with Ser26 and several Van der Waals forces with its lateral chain. Those compounds of the **S7** group, containing phenyl ethanol as substituent, formed H-bonds and electrostatic interactions with Phe20 or Asp23, respectively. In the case of **F4S7-m/-p** an electrostatic interaction between the NH^+^ group and the carboxyl group of His14 was observed to happen in an analogous way to what occurs in methanamine compounds (**S3**-**m/-p** family) in which H-bonds and electrostatic interactions with Asp23 and Phe20 are stablished. However, none of **F4S7-m/-p** showed better ΔG values than the methanamine substituent (**S4**) in which interaction between Asp23, Glu22 and the NH_2_ group are observed leaving the aromatic ring or the NH^+^ group available to establish π-π or π-cation stacking with Phe19 and Phe20. This feature could not be seen in **F1S4-m/p** compounds due to distancing between NH^+^ group with Phe19/20 or Asp23/Glu22 carboxylic acid groups measuring 10.5/12.5 and 7.4/10.2 Ǻ, respectively. Similar distancing features were observed in the **F4S4-m/p** for Asp23/Glu22 lateral chains with the aromatic eter contained in the morpholine group. Otherwise, the **F2S4** and **F3S4 -m** or **-p** compounds presented similar interactions with distancing ranges varying from 1.5 to 3.5 Å for the NH_2_ methanamine group with the lateral chain of Asp23 and Glu22; and 4–10 Ǻ with lateral chain of Phe19 or Phe20 with NH^+^ or aromatic ring (Fig 2C–2F).

**Fig 3:**
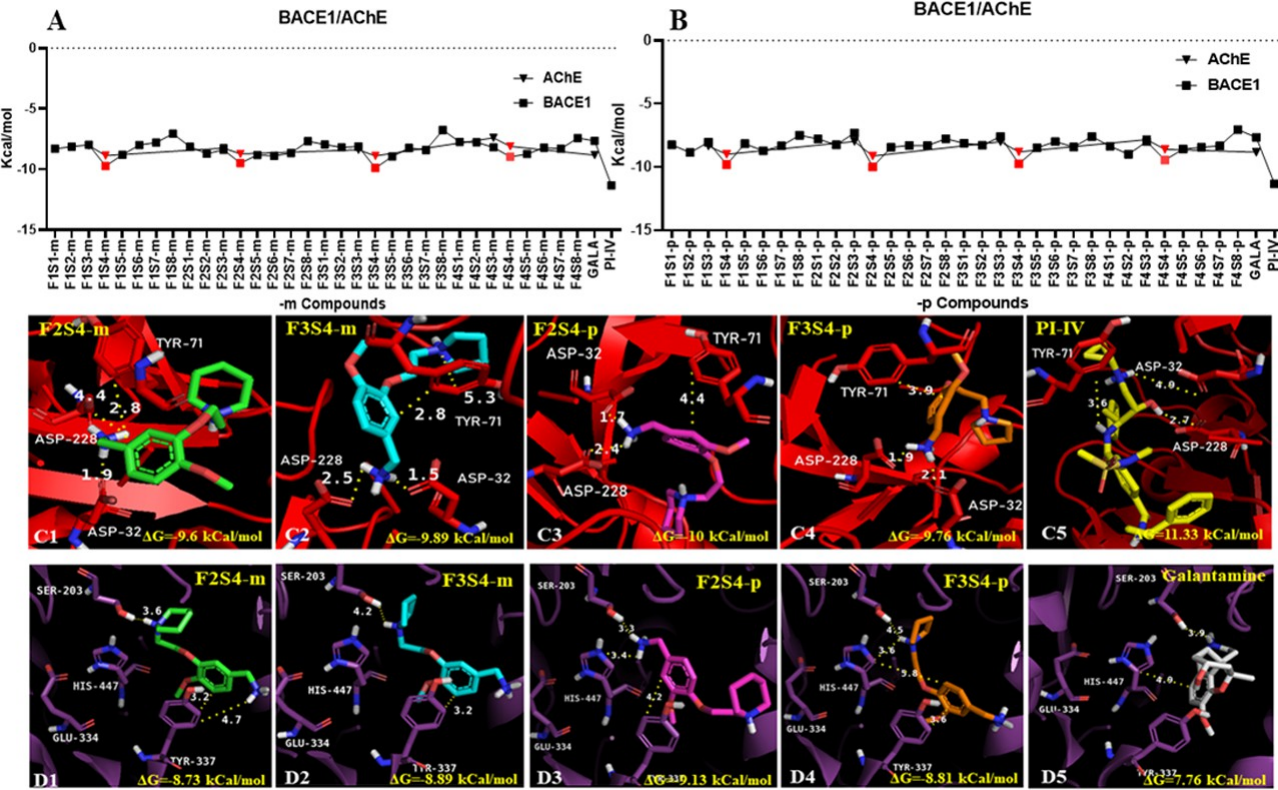
Docking results of design and known compounds towards BACE1 and human AChE. Free energy values obtained for: **A)** 1,3 substituted **(-meta)** and **B)** 1,4 substituted **(-para)** compounds toward BACE1(**squares**) and AChE (**triangles**). The methanamine (**S4**) bearing compounds (red squares and triangles) showed the best values towards the two proteins, beating galantamine but do not **IP-IV**. Binding analysis of containing methanamine compounds (**S4**) and PI-IV toward: **C1-5)** BACE1 (red) and **D1-5**) human AChE.

The results from the nonbond interaction between all compounds with BACE1 are shown in S2 Table in S1 File. The lowest free energy values obtained for **F2S4-p**, **F3S4-m**, **F1S4-p**, **F3S4-p**, **F1S4-m,** and **F2S4-m** were -10, -9.89, -9.81, -9.76, -9.73 and -9.5 Kcal/mol respectively (Fig 3A and 3B). These values are like those reported elsewhere for a series of PDB-based ligands against AChE, BACE1 and γ-secretase among other promising drug-targeted enzymes for the AD treatment [37–39]. The best results obtained from the docking interaction between ligands and Aβ_1–42_ were obtained for compounds **F2S4-m / p** and **F3S4-m**, therefore, we focused on describing their binding mode to BACE1.

Regarding the binding mode analysis towards BACE1 **F2S4-m** (Fig 3C1), **F3S4-m** (Fig 3C2), **F2S4-p** (Fig 3C3) and **F3S4-p** (Fig 3C4) compounds were able to reach the binding pocket where the aspartic dyad is located at 2–3 Ǻ due to an electrostatic interaction with the amine groups of compounds. In the case of peptidomimetic inhibitor (Fig 3C5), only H-bonds interactions could be established by its hydroxyl group.

Likewise, compounds **F3S4-m**, **F3S4-p** and **F2S4-m** have in common the establishment of at least one of the following type of interactions; π-σ, π-cation or π-π between the aromatic or polar rings of the molecule in question with Tyr71 (Fig 3C1–3C3). These types of interactions agree with those reported elsewhere in which phenolic compounds interact with amino residues of AChE [40]. On the other hand, compound **F2S4-p**, which showed the lowest free energy values, establishes an intramolecular π-cation interaction causing the NH^+^ group approaching to Gly230 and establishing an H-bonding interaction. The polar groups of **IP-IV** were unable to establish electrostatic interactions with the aspartic dyad or other acidic residue; instead, hydrogen bond interactions between Gly74, Thr232, Phe108 Asp228 and the amino, carbonyl, sulfonamide and hydroxyl groups of **IP-IV** were observed, respectively (Fig 3C5).

Therefore, those molecules designed with an extra amino group were selected, corresponding to the substituents **S3-m/p** and **S4-m/p** of the four families of compounds to be explored by docking studies on human AChE. The best results obtained were those from compounds with methanamine (**S4**) substituent over those containing methanamine (**S3**) (Fig 3A and 3B). Four of the best compounds were **F2S4-p**, **F3S4-m**, **F3S4-p,** and F**2S4-m** with free energy values of -9.13, -8.89, -8.81 and -8.73 kcal/mol respectively, being these values better than the control compound galantamine with a free energy value of -7.66 kcal/mol.

Analyzing the binding mode of **F2S4-m** (Fig 3D1), **F3S4-m** (Fig 3D2), **F2S4-p** (Fig 3D3), **F3S4-p** (Fig 3D4) and galantamine (Fig 3D5) towards the AChE binding pocket, it is observed that none of them had the ability to reach Glu334 (S2 Table in S1 File). However, all the designed compounds were placed at 3–5 Ǻ to establish π-π or π-cation interactions with the aromatic ring of Tyr337. It is observed that despite not having the ability to interact with Glu334, several π-π, π-σ, π-cation or electrostatic interactions with different polar and aromatic residues were established.

**F2S4-m** and **F3S4-m** established π-π interactions with Trp86 and Tyr337 through their aromatic ring, π-σ with Trp86 and Tyr337 with the two ether groups and π-cation with Trp86 through their heterocyclic amines, as well as Asp74, His447 and Glu202 and numerous H-bonds with them. Compound **F2S4-p** established the same type of interactions as the other two compounds, with the addition of a π-π and π-cation interaction with His447 through its aromatic group and NH_2,_ respectively.

### 3.2. The compounds with methanamine substituent complying with all Lipinsky rules and can cross the BBB predicted by *in silico* studies

The molecules with better results from molecular docking (ΔG and binding mode) and the reported inhibitors employed as controls were submitted to physicochemical properties evaluation using OSIRIS property explorer and molinspiration server. The results from all compounds according to the Lipinsky rules (MW < 500 g /mol, Logp<5, H-bonds donator < 5 and H-bond acceptor <10) are presented in Table 1. All molecules evaluated from the **S4** series (**F1S4**, **F2S4**, **F3S4** and **F2S4**) both in *meta* and *para* substitution position complied with all the Lipinsky rules, this outcome allowed us to hypothesize that these compounds would have a good behavior and bioavailability at a systemic level after oral administration. In computational prediction of biological effects none of the compounds shown properties as tumorigenic or mutagenic, **F3S4-m** shown medium risk as a teratogenic due to the presence of *N*-(-2-hydroxyethyl)-pyrrolidine. Furthermore, the *in silico* BBB permeability predictors, showed that all compounds from the S4 family can cross the BBB, showing that curcumin and the *PI-IV* inhibitor not. It is important to mention that these parameters were calculated considering the negative and/or positive ionization of the molecule at physiological pH.

**Table 1:** *In silico* properties, biological activity prediction and Blood Brain Barrier (BBB) permeability prediction Log P (bipartition coefficient) MW (molecular weight); nON, (number of hydrogen bond acceptors); nOHNH (number of hydrogen bond donors); Mt (mutagenicity); Tg, (teratogenicity), T (tumorigenic).

	*Physicochemical properties*							*BBB permeability prediction*			
*Compound*	*LogP*	*MW*	*nON*	*nOHNH*	*M*	*T*	*Tg*	BBB predictor server		Swiiss ADME	Light BBB
								Score	Result	Result	Result
***F1S4-m***	**1.19**	**250.34**	**4**	**2**	**-**	**-**	**-**	**0.081**	**+**	**+**	**+**
***F2S4-m***	**1.71**	**264.37**	**4**	**2**	**-**	**-**	**-**	**0.081**	**+**	**+**	**+**
***F3S4-m***	**1.2**	**250.34**	**4**	**2**	**-**	**-**	**M**	**0.088**	**+**	**+**	**+**
***F4S4-m***	**0.64**	**266.34**	**5**	**2**	**-**	**-**	**-**	**0.088**	**+**	**+**	**+**
***F1S4-p***	**1.19**	**250.34**	**4**	**2**	**-**	**-**	**-**	**0.092**	**+**	**+**	**+**
***F2S4-p***	**1.71**	**264.37**	**4**	**2**	**-**	**-**	**-**	**0.092**	**+**	**+**	**+**
***F3S4-p***	**1.2**	**250.34**	**4**	**2**	**-**	**-**	**M**	**0.079**	**+**	**+**	**+**
***F4S4-p***	**0.64**	**266.34**	**5**	**2**	**-**	**-**	**-**	**0.079**	**+**	**+**	**+**
***Galantamine***	**1.54**	**287.36**	**4**	**1**	**-**	**-**	**-**	**0.142**	**+**	**+**	**+**
***Curcumin***	**2.3**	**368.38**	**6**	**2**	**-**	**-**	**-**	**0.017**	**-**	**-**	**-**
***PI-IV***	**2.98**	**578.74**	**9**	**4**	**-**	**-**	**-**	**0.053**	**+**	**-**	**-**

### 3.3. Compound F2S4-m inhibit the Aβ_1–42_ oligomerization better than F2S4-p and F3S4-m compounds

**Fig 4:**
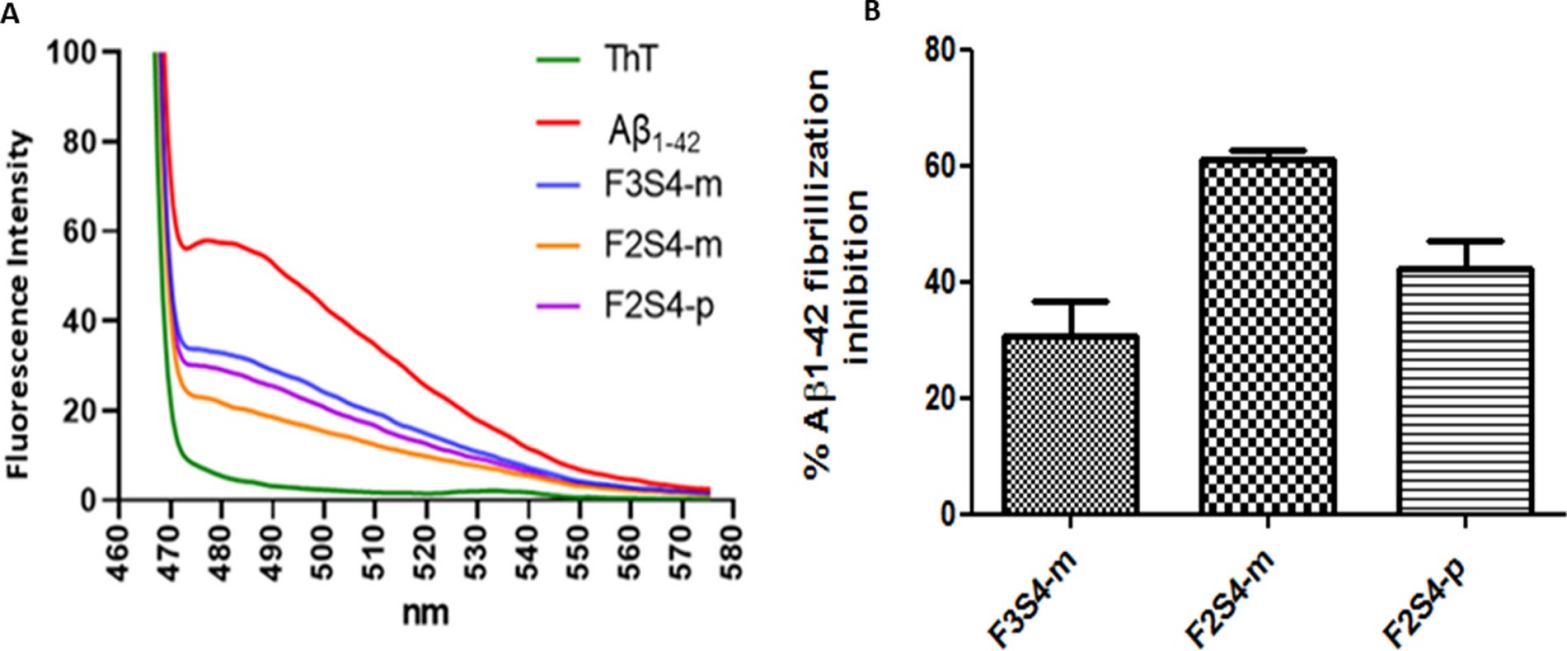
Percentage of inhibition of Aβ_1–42_ fibrillation. A) ThT assay using compounds **F2S4-m**, **F2S4-p,** and **F3S4-m** at 100μM B) Plot of percentage of inhibition of Aβ_1–42_ fibrillation by the compounds.

The inhibition of Aβ_1–42_ oligomerization results is shown in Fig 4. The ThT intensity when is bonded to fibrillated Aβ_1–42_ is shown in Fig 4A. The highest intensity is observed because the presence of Aβ_1–42_, a reduction in intensity indicates that fibrillated Aβ_1–42_ has been diminished by the presence of the evaluated compounds. The fluorescence intensity was determined as shown in Fig 4A, the mean of percentage of Aβ_1–42_ fibrillization was obtained and plotted in Fig 4B, where is possible to observe that **F3S4-m** inhibited in 30.5%, **F2S4-p** in 42.1%, and **F2S4-m** in 60.9% the Aβ_1–42_ fibrillization. The average of the inhibition percentages with their respective standard error is shown in Fig 4.

### 3.4. F3S4-m has better BACE1 inhibition activity than F2S4-m and F2S4-p

**Fig 5:**
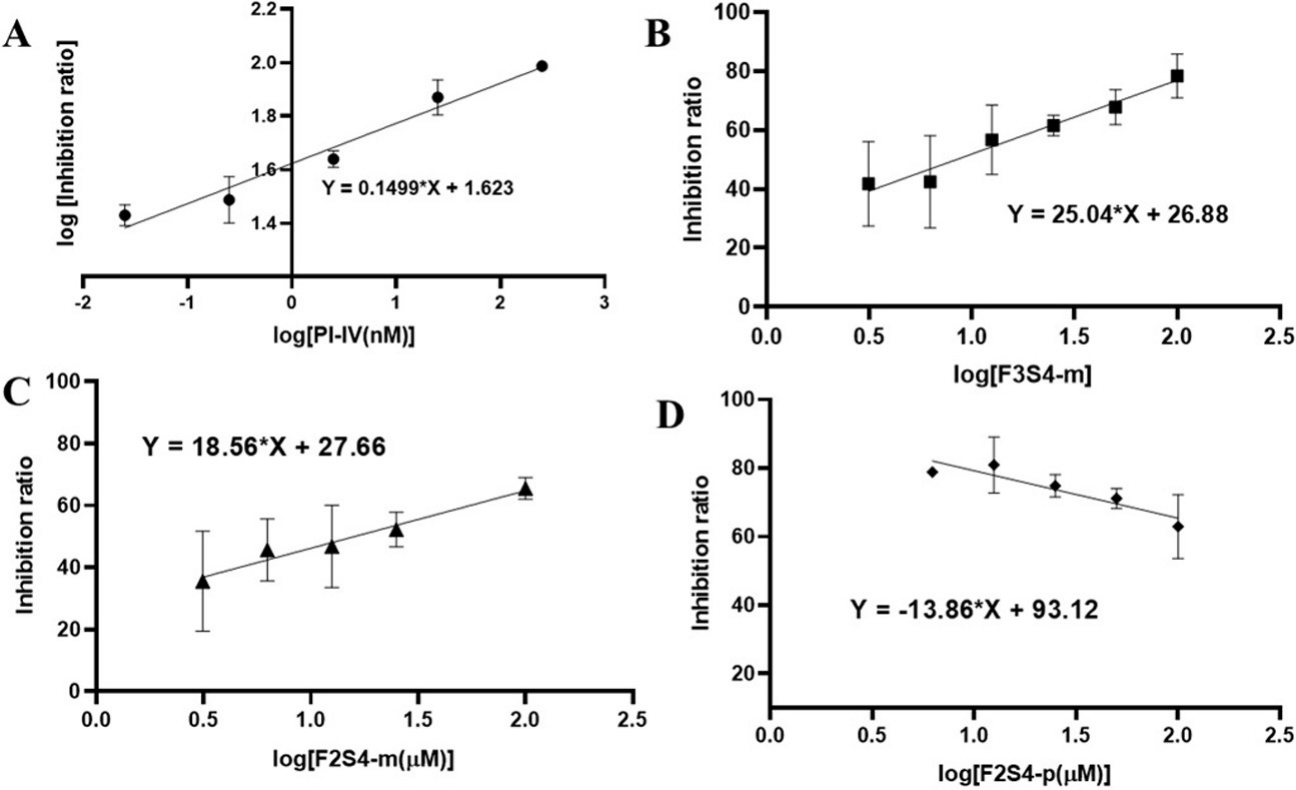
Inhibition of the BACE1 enzyme by: (A) known inhibitor IV, (B) **F3S4-m**, (C) **F2S4-m** and (D) **F2S4-p**. The standard error was calculated from the fluorescence measurements of the duplicate of two independent assays with n = 3.

The IC_50_ values for the BACE1 inhibition were obtained from linear regression plots using the logarithmic function being plotting the % inhibition against log [inhibitor (μM)] or log % inhibition against log [inhibitor (μM)] (Fig 5A). The double logarithm function is done to fit the data to a straight line since, as the inhibitor is more powerful, the data would not fit if we only plotted the log of the concentrations.

For **F2S4-m**, **F3S4-m,** and **F2S4-p**, the IC_50_ was obtained from linear regression plot of inhibition ratio against the log of inhibitor concentration, Fig 5B and 5C. Then, substituting in the plot equation, when the enzyme has the 50% of inhibition the concentration corresponding to the IC_50_ values was determined as: 3.20 nM for PI-IV, 8.38 μM and 15.97 μM for **F3S4-m** and **F2S4-m**, respectively. Compound **F2S4-p** showed a proportional relationship between its concentration and the fluorescence emission; or, inversely proportional to the inhibition of BACE1 enzyme (Fig 5D). Therefore, it was not possible to determine its IC_50_ value.

### 3.5. F3S4-m has better AChE inhibition activity than F2S4-m and F2S4-p

**Fig 6:**
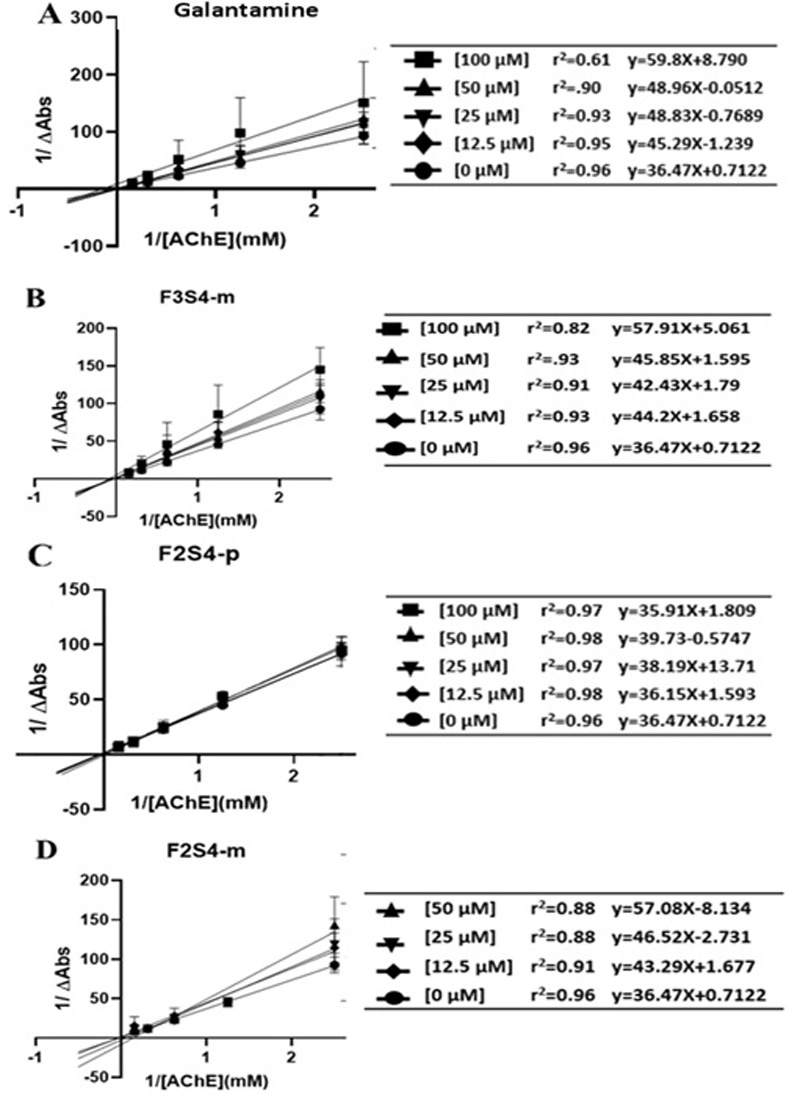
Lineweaver-Burk kinetic model of AChE inhibition by A) Galantamine, B) F3S4-m, C) F2S4-p and D) F2S4-m.

A Lineweaver Burk representation was made by plotting [1/ΔAbs] versus [1/ACh] for each case; Galantamine (Fig 6A), **F3S4-m** (Fig 6B), **F2S4-p** (Fig 6C) and **F2S4-m** (Fig 6D). As can be seen in Fig 6C compound **F2S4-p** showed no significant effect as an AChE inhibitor.

**Fig 7:**
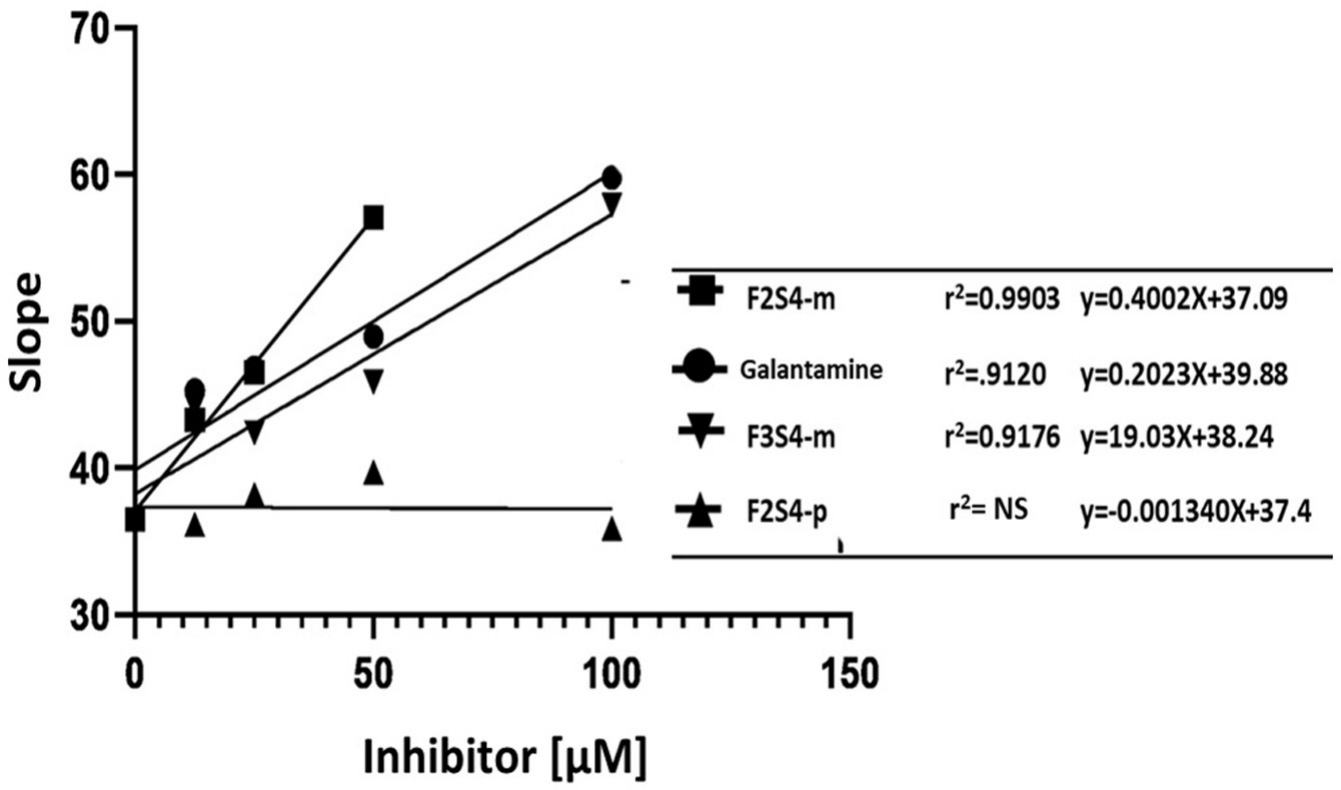
Determination of the inhibition constant (Ki) of compounds F2S4-m, F3S4-m, F2S4-p and galantamine by graphing the slope of lineaweaver-burk model against the respective inhibitor concentration.

Subsequently, the graphic determination of the inhibition constant (Ki) was carried out by means of the expression of the slopes obtained from the representations of double reciprocals as a function of the concentration of each inhibitor. Ki (Galantamine) = 0.20 μM, Ki (**F2S4-p**) = NS, Ki (**F2S4-m**) = 0.40 μM, Ki (**F3S4-m**) = 0.19 μM (Fig 7) were obtained. The compound with similar Ki to galantamine was **F3S4-m**.

### 3.6. F2S4-p was more cytotoxic than F2S4-m and F3S4-m evaluated in cell cultures

**Fig 8:**
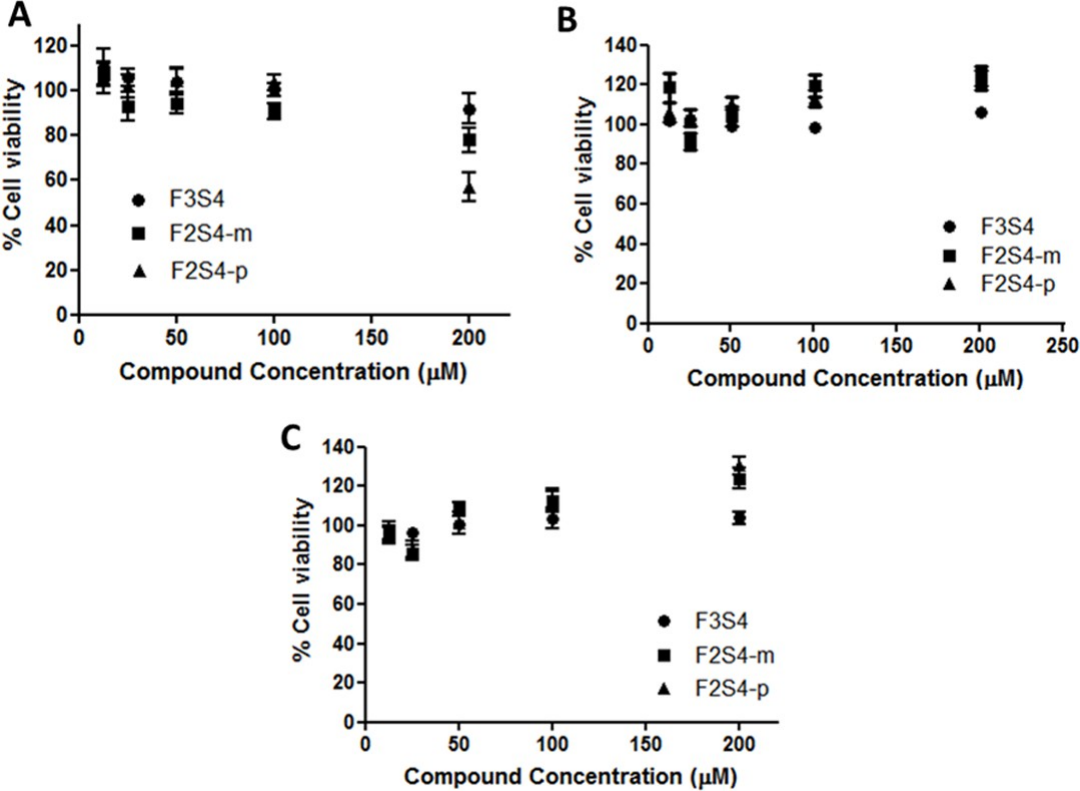
Cytotoxic evaluation of compounds **F3S4-m**, **F2S4-m**, **F2S4-p** in different cell lines by MTT assay: A) Fibroblasts, B) Primary culture of astrocytes and C) PC12 cells.

The cytotoxicity of compounds was evaluated in fibroblast cells, astrocyte and PC12 cell cultures. As can be seen in Fig 8, only compound **F2S4-p** showed cytotoxic in fibroblast cultures but not in astrocyte cultures, neither in PC12 cell cultures (Fig 8B and 8C). Compounds **F3S4-m** and **F2S4-m** were not cytotoxic in any of the evaluated cell lines up to 200 μM.

## 4. Discussion

Even though, in recent years numerous advances have been made to understanding the pathological mechanisms involved in AD, to date there is no treatment capable of stopping the course of the disease [41] for that reason, it is important to continue the search for new molecules that can be able to recognize and inhibit more than one target of the pathological processes causing AD [42]. Given that the amyloidogenic hypothesis continues to be one of the main accepted hypotheses, it is important to focus on the design and use of new compounds towards molecules involved with this pathway [43]. Thus, the BACE1 and aggregation of Aβ_1–42_ in oligomers and fibrils have become important pharmacological targets since the production and aggregation of Aβ_1–42_ can cause neuronal death [44]. Accumulation of Aβ_1–42_ is also related to cholinergic deficit [28, 45]. Therefore, the AChE is also one of the main therapeutic targets in AD, because its inhibition increases ACh levels and thus retards cognitive deficit [46].

Therefore, it is of foremost importance to design compounds that may have inhibitory activity on AChE, BACE1 and decrease the aggregation of Aβ_1–42_, all these biomolecules involved in AD [44]. Consequently, in this study there were designed new compounds considering the presence of a heterocyclic tertiary amine capable of protonation at physiological pH and other chemical moieties capable to establish electrostatic interactions, hydrogen bonding, hydrophobic interactions as well as π-π interactions with the aminoacid residues located inside the catalytic site of the targets.

Results from *in silico* studies confirmed that **F3S4-m**, **F2S4-m,** and **F2S4-p** established at least one polar interaction with one of the aa residues from the active sites in the three targets employed. Regarding the Aβ_1–42_ aggregation, compounds were able to stablish nonbond interaction with Asp23 and Glu22 which prevents the interaction of this residues with Lys 28 leading to blockage of the subsequent Aβ_1–42_ aggregation. However, the presence of a methylene bound to the heterocyclic amine (Family F1) seems to keep the NH^+^ group away from the acid dyad and the Phe19 and Phe20 residues based on the analysis of the pyrrolidine added compounds (**F1S4-m** and **F3S4-m**), a considerable difference in their free energy was found due to was better **F3S4-m** than **F1S4-m**. There are reported studies about the binding capacity of nicotine and two of its main fragments: pyridine and methyl pyrrolidine, towards Aβ_1–28_ in its monomeric form with a remarkable inhibition of β-folded formation and precipitation based on interaction with His residues [47]. Therefore, the interaction of pyridine and methyl pyrrolidine could be different depending on the Aβ length as in Aβ_1–28_ the acid dyad is the terminal carboxyl in the peptide and the folding in the Aβ_1–28_ is different from Aβ_1–42_.

The presence of a -NH_2_ group at the aromatic ring allows the molecule to be placed between acidic residues in Aβ_1–42_ without distancing away the pyrrolidine from the acid dyad or from the aromatic rings of Phe19 and Phe20 in Aβ_1–42_, allowing the establishment of π-cation interactions; for this reason the presence of three atoms of distance between the aromatic ring and the amine heterocycle seems to be fundamental to maintain the amine within < 6 Å of interaction with the Phe ring.

There are chemical differences and similarities between **S4** and **S3** family compounds, both containing diamino groups located at the same distance; is the presence of an extra alkyl group decreasing its basicity and therefore the ability to favor the reaction with the acid dyad. The size of compounds does not seem to be a limitation to reach the active site of BACE1 or Aβ_1–42_, at least in its monomeric form. In the same way, the docking studies performed with BACE1, the *meta*-substituted amine group of molecules showed better qualities allowing to place the molecule in the middle of the catalytic dyad forming electrostatic interactions, leaving the heterocyclic amine available to establish extra polar interactions with Asp228 at 5–6 Å and cation-π with Tyr71 at 6Å distance or even the aromatic ring with Tyr71 through α-π interactions. In addition, the compounds with pyrrolidine and the amine group in the aromatic ring were the best compounds for AChE inhibition, having less ΔG values means favored affinity on the enzyme.

It was observed an outstanding performance of all compounds in the *in silico* pharmacokinetic studies, complied with the Lipinsky’s rule. Although the **F3S4-m** compound showed the lowest free energy by docking studies, possible teratogenic effects were observed due to the presence of N-(2-hydroxyethyl)-pyrrolidine. Nevertheless, this substituent can be found in drugs already approved for *in vivo* use such as Levetiracetam and brivaracetam diclofenamic acid [48]. In addition, among the all three synthesized compounds, **F2S4-m** demonstrated to reduce by 60.9% the amount of fibrillated Aβ_1–42_ by ThT assay.

**F2S4-m** and **F3S4-m** showed inhibitory activity against AChE and BACE1 due to these molecules have an amine group in their structures like donepezil, a current AChE inhibitor in the market [49]. However, the results obtained suggest that the amine position within the molecule is relevant to the inhibitory activity, due to **F2S4-p** compound did not show inhibitory activity on the tested enzymes.

Additionally, few studies suggest that pyrrolidine could be better group to inhibit AChE than piperidine, however, other studies have even focused on the design of 1,3-linked benzylpiperidine compounds, dimethylbenzimidazoline as cholinesterase inhibitors of both AChE and BChE [50, 51]. In addition, some multitarget therapeutic strategies for AD proposed dual compounds that would allow the inhibition of ChE and BACE1 or that can inhibit ChE and Glycogen synthase kinase-3β (GSK-3β) or ChE and monoamine oxidase (MAO) enzyme, and other combinations of compounds that may influence ChE and receptors. But it is not mentioned that they inhibit the Aβ aggregation, so the compounds designed here identified from the chemical characteristics of the active site of three important targets in AD could be a multitarget tool on more than one pharmacological target in the AD [10, 44]. In addition, **F3S4-m**, **F2S4-m** are not cytotoxic in the PC12, astrocytes and fibroblast, the compound **F2S4-p** shows cytotoxic effect against astrocytes cells. Furthermore, more experiments need to be done to explore the toxicity of **F3S4-m** as *in silico* analyses showed that it might has teratogenic effects.

Therefore, the compounds herein designed and obtained could be a good promise for the treatment of AD as the combination of the pharmacophore groups in a small molecule could be important to reach the brain. As these compounds have a small molecular weight, they can be incorporated in different nanotechnology-based drug delivery approaches (e.g., functionalized nanoparticles, nano micellar water formulations, lipid-based nanoparticles, nanogels, etc.) to help crossing the BBB and reach directly into the brain [52].

## 5. Conclusion

Newly developed compounds **F2S4-m** and **F3S4-m** are proposed as potential multitarget therapeutics for the AD as these molecules have shown to inhibit three important pharmacological targets in the AD, Aβ_1–42_ aggregation, and AChE and BACE1 enzyme activities *in vitro*. Despite compound **F2S4-p** did not show inhibition of AChE and BACE1 activity, but it showed Aβ_1–42_ aggregation, this molecule could be employed in combination with any of the compounds as an interesting approach employing different mechanisms of action as a more effective way to tackle a complex neurological disease.

## Supporting information

S1 File Supplementary material V-1-2. Tables of free energy values (kcal/mol) and spectroscopic characterisation of synthesised compounds.
(DOCX)
