# Supplementary material for: New compounds from heterocyclic amines scaffold with multitarget inhibitory activity on Aβ aggregation, AChE, and BACE1 in the Alzheimer disease
Source: PLoS One. 2022 Jun 3;17(6):e0269129. doi: 10.1371/journal.pone.0269129 (PMC9165844; doi:10.1371/journal.pone.0269129)
Supplement: S1 File — Tables of free energy values (kcal/mol) and spectroscopic characterisation of synthesised compounds. (DOCX) [file pone.0269129.s001.docx]

**S1 File**

**NEW COMPOUNDS FROM HETEROCYCLIC AMINES SCAFFOLD WITH MULTITARGET INHIBITORY ACTIVITY ON Aβ AGGREGATION, AChE, AND BACE1 IN THE ALZHEIMER DISEASE**

Iohanan Daniel García Marín ^#a^, Raúl Horacio Camarillo López ^#a^, Oscar Aurelio Martínez, Itzia Irene Padilla-Martínez ^b^_,_ José Correa-Basurto^c^, Martha Cecilia Rosales-Hernández ^a*^.

**S1 Table.** Free energy values (kcal/mol) and amino acids able to interact in a 5 Ǻ radius toward different amyloid-β conformations.

| Compound | α-Helix. | | β-Sheet | | Random Coil (RC) | |
| --- | --- | --- | --- | --- | --- | --- |
| F1S1-m | **-4.7** | ASN27, LYS28, ALA30, ILE31,  MET35, VAL40, ILE41, ALA42 | **-4.65** | ALA21, GLU22, ASP23, VAL24, GLY25, SER26, ILE32, LEU34 | **-5.97** | PHE19, GLU22, ASP23, VAL24, SER26, ASN27, LYS28 |
| F1S2-m | **-5.44** | TYR10, GLU11, VAL12, HIS13, GLN15,  LYS16, PHE19, ASP23 | **-4.84** | ALA21, GLU22, ASP23, VAL24, GLY25, ILE32, LEU34 | **-5.37** | GLU22, ASP23, VAL24, GLY25, SER26, ASN27, LYS28 |
| F1S3-m | **-5.71** | GLN15, PH19, PHE20, ASP23,  VAL24, ASN27. | **-4.95** | ASP23, VAL24, GLY5, SER26, LYS28, ILE31, ILE32, GLY33, LEU34, MET35 | **-5.32** | PHE19, GLU22, ASP23, VAL24, GLY25, SER26, ASN27, LYS28 |
| F1S4-m | **-7.24** | GLN15, PH19, PHE20, ASP23, VAL24, ASN27. | **-6.11** | ASP23, VAL24, GLY25, ILE31, ILE32, GLY33, LEU34, MET35. | **-6.08** | HSD13, HSD14, LEU17, VAL18, ALA21, GLU22 |
| F1S5-m | **-5.62** | GLU11, VAL12, GLN15, PHE19, PHE20, ASP23, ASN27 | **-4.99** | ALA21, GLU22, ASP23, VAL24, GLY25, ILE32, LEU34, VAL36 | **-4.66** | HSD14, GLN15, VAL18, PHE19, GLU22, ASP23 |
| F1S6-m | **-5.22** | PHE19, PHE20, ASP23, VAL24, ASN27, LYS28, ILE31 | **-4.75** | ALA21, GLU22, ASP23, VAL24, GLY25, ILE32, LEU34, VAL36 | **-5.35** | PHE19, GLU22, ASP23, VAL24, SER26, ASN27, LYS28 |
| F1S7-m | **-5.67** | TYR10, GLU11, VAL12, HIS13, GLN15, LYS16, PHE19, ASP23, ASN27 | **-5.24** | ASP23, VAL24, GLY25, SER26, LYS28, ASN27, GLY29, ILE31, ILE32, GLY33, LEU34, MET35 | **-5.04** | PHE19, GLU22, ASP23, VAL24, GLY25, SER26, ASN27, LYS28 |
| F1S8-m | **-4.57** | VAL12, GLN15, LYS16, LEU17, PHE19, PHE20 | **-4.01** | PHE19, PHE20, ALA21, GLU22, VAL36 | **-5** | ASP23, VAL24, GLY25, SER26, ASN27, LYS28. |
| F2S1-m | **-4.93** | HIS14, VAL18, ALA21, GLU22, SER26 | **-5.14** | ALA21, GLU22, ASP23, VAL24, GLY25, SER26, ASN27, LYS28, GLY29, ILE32, LEU34 | **-5.89** | GLU22, ASP23, VAL24, GLY25, SER26, ASN7, LYS28 |
| F2S2-m | **-5.7** | ALA21, GLU22, VAL24, GLY25, SER26 | **-5.27** | ALA21, GLU22, ASP23, VAL24, GLY25, ILE32, LEU34 | **-5.04** | GLU11, VAL12, HSD14, GLN15, LYS16, VAL18, PHE19, PHE20, ALA21, GLU22, ASP23, VAL24, GLY25, MET35 |
| F2S3-m | **-5.88** | GLN15, VAL18, PHE19, PHE20, ALA21 , GLU22, ASP23, VAL24, ASN27 | **-5.01** | GLU22, ASP23, VAL24, GLY25, ILE32, LEU34 | **-5.19** | GLU22, ASP23, VAL24, GLY25, SER26, ASN27, LYS28 |
| F2S4-m | **-7.35** | GLN15, VAL18, PHE19, PHE20, GLU22, ASP23. VAL24. SER26, ASN27. | **-5.78** | PHE20, ALA21, GLU22, ASP23, VAL24 | **-5.86** | VAL18, ALA21, GLU22, ASP23, VAL24, SER26 |
| F2S5-m | **-5.93** | GLN15, VAL18, PHE19, PHE20, GLU22, ASP23, VAL24, ASN27, LYS28 | **-4.95** | PHE19, PHE20, ALA21, GLU22, ASP23, GLY33, LEU34, MET35, VAL36, GLY37, GLY38, VAL39 | **-4.05** | PHE19, GLU22, ASP23, VAL24, GLY25, SER26, ASN27, LYS28 |
| F2S6-m | **-5.79** | GLN15, VAL18, PHE19, PHE20, ALA21, GLU22, ASP23, ASN27 | **-5.42** | ALA21, GLU22, ASP23, VAL24, GLY25, SER26, ASN27, LYS28, GLY29, ILE32, LEU34 | **-5.37** | PHE19, GLU22, ASP23, VAL24, GLY25, SER26, ASN27, LYS28 |
| F2S7-m | **-5.3** | GLN15, VAL18, PHE19, PHE20, ALA21, GLU22, ASP23, ASN27 | **-4.76** | GLU22, ASP23, VAL24, GLY25, SER26, ILE32, LEU34 | **-4.53** | PHE19, GLU22, ASP23, VAL24, GLY5, SER26, ASN27, LYS28 |
| F2S8-m | **-5.09** | VAL12, GLN15, LYS16, LEU17, PHE19, PHE20 | **-3.88** | GLU22, ASP23, VAL24, GLY25, ILE32, LEU34 | **-5.38** | ASP23, VAL24, GLY25, SER26, ASN27, LYS28. |
| F3S1-m | **-4.73** | ASP23, ASN27, LYS28, ILE31, ILE41, ALA42- | **-4.93** | ALA21, GLU22, ASP23, VAL24, GLY25, ILE32, LEU34 | **-5.86** | PHE19, ASP23, VAL24, SER26, ASN27, LYS28 |
| F3S2-m | **-5.87** | TYR10, GLU11, VAL12, HIS13, HIS14,   GLN15, LYS16, VAL18, PHE19, GLU22, ASP23, ASN27 | **-5.09** | GLU22, ASP23, VAL24, GLY25, SER26, ILE32, GLY33, LEU34, MET35 | **-5.03** | PHE19, ASP23, VAL24, GLY25, SER26, ASN27, LYS28 |
| F3S3-m | **-5.47** | GLN15, VAL18, PHE19, PHE20, ALA21, GLU22, ASP23, VAL24, ASN27 | **-4.67** | ASP23, VAL24, GLY25, , SER26, ASN27, LYS28, GLY29, ILE32, GLY33, LEU34 | **-5.12** | PHE19, GLU22, ASP23, VAL24, GLY25, SER26, ASN27, LYS28 |
| F3S4-m | **-7.44** | GLN15, PHE19, PHE20, GLU22, ASP23, ASN27 | **-6.06** | PHE20, ALA21, GLU22, ASP23, VAL24. | **-5.71** | VAL18, ALA21, GLU22, ASP23, VAL24, SER26 |
| F3S5-m | **-5.42** | TYR10, GLU11, HIS14, GLN15, VAL18, PHE19, PHE20, GLU22, ASP23, ASN27 | **-4.84** | PHE19, PHE20, ALA21, GLU22, ASP23, VAL24, LEU34, MET35, VAL36 | **-4.2** | LEU17, VAL18, PHE20, ALA21, GLU22, GLY25, SER26, LYS28, GLY29, ALA30, ILE31 |
| F3S6-m | **-5.29** | GLN15, VAL18, PHE19, PHE20, GLU22, ASP23, VAL24, ASN27, LYS 28 | **-4.59** | PHE19, PHE20, ALA21, GLU22, ASP23, VAL24, LEU34, MET35, VAL36 | **-5.29** | PHE19, GLU22, ASP23, VAL24, SER26, ASN27, LYS28 |
| F3S7-m | **-5.28** | GLN15, VAL18, PHE19, PHE20, GLU22, ASP23, VAL24, ASN27, LYS 28 | **-4.69** | ALA21, GLU22, ASP23, VAL24, GLY25, ILE32, LEU34 | **-4.58** | PHE19, GLU22, ASP23, VAL24, GLY25, SER26, ASN27, LYS28 |
| F3S8-m | **-4.64** | VAL12, GLN15, LYS16, PHE19, PHE20 | **-3.52** | ALA21, GLU22, ASP23, VAL24, GLY25, LEU34, VAL36 | **-4.41** | ASP23, VAL24, SER26, ASN27, LYS28 |
| F4S1-m | **-4.79** | ASP23, ASN27, LYS28, ILE31, ILE41, ALA42. | **-4.79** | ALA21, GLU22, ASP23, VAL24, GLY25, SER26, LYS28, ILE32, LEU34. | **-5.58** | PHE19, ASP23, VAL24, GLY25, SER26, ASN27, LYS28 |
| F4S2-m | **-5.09** | ALA21, GLU22, VAL24, GLY25, SER26 | **-4.61** | GLU22, ASP23, VAL24, ILE32, GLY33, LEU34, MET35, VAL36. | **-4.82** | PHE19, ASP23, VAL24, SER26, ASN27, LYS28 |
| F4S3-m | **-5.56** | HIS14, VAL18, ALA21, GLU22, SER26 | **-4.79** | GLU22, ASP23, VAL24, GLY25, SER26, ILE32, LEU34 | **-4.73** | PHE19, GLU22, ASP23, VAL24, GLY25, SER26, ASN27, LYS28 |
| F4S4-m | **-6.93** | GLN15, VAL18, PHE19, PHE20, GLU22, ASP23, VAL24, SER26, ASN27 | **-5.43** | PHE19, OHE20, ALA21, GLU22, ASP23, VAL24, LEU34 | **-5.62** | VAL18, GLU22, ASP23, VAL24, SER26. |
| F4S5-m | **-5.22** | HIS14, VAL18, ALA21, GLU22, SER26 | **-4.33** | PHE19, PHE20, ALA21, GLU22, ASP23, VAL24, GLY25, ILE32, LEU34, VAL36. | **-4.08** | HSD14, GLN15, VAL18, PHE19, ASP23, VAL24. |
| F4S6-m | **-5.17** | ASP7, TYR10, GLU11, VAL12, HIS13,  HIS14, GLN15, LYS16, VAL18, PHE19,  PHE20, GLU22, ASP23, ASN27 | **-5.12** | ALA21, GLU22, ASP23, VAL24, GLY25, LYS28, GLY29, ILE32, LEU34 | **-4.78** | PHE19, GLU22, ASP23, VAL24, GLY25, SER26, ASN27, LYS28 |
| F4S7-m | **-5.09** | HIS14, VAL18, ALA21, GLU22, SER26 | **-4.43** | PHE20, ALA21, GLU22, ASP23, VAL24, GLY33, LEU34, MET35 | **-4.24** | PHE19, GLU22, ASP23, VAL24, GLY25, SER26, ASN27, LYS28 |
| F4S8-m | **-4** | GLU11, VAL12, HIS14, GLN15, PHE19, PHE20, ASP23, ASN27 | **-3.59** | GLU22, ASP23, VAL24, GLY25, ILE32, LEU34 | **-4.69** | ASP23, VAL24, GLY25, SER26, ASN27, LYS28. |
| F1S1-p | **-4.8** | HIS13, HIS14, GLN15, LYS16, LEU17, PHE19, PHE20, ALA21, GLU22, ASP23, VAL24, ASN27, LYS28, ILE31, ILE41 | **-4.6** | ASP23, VAL24, GLY25, SER26, LYS28, ASN27, GLY29, ILE31, ILE32, GLY33, LEU34, MET35 | **-4.91** | ASP23, VAL24, SER26, ASN27, LYS28 |
| F1S2-p | **-6.32** | GLU11, VAL12, HIS14, GLN15, LYS16, VAL18, PHE19, GLU22, ASP23, ASN27 | **-5.46** | ASP23, VAL24, GLY25, LYS28, ILE32, GLY33, LEU34, MET35, VAL36- | **-4.94** | LEU17, VAL18, ALA21, GLU22, ASP23, GLY25, SER26 |
| F1S3-p | **-5.05** | GLU11, HIS14, GLN15, VAL18, PHE19, PHE20, GLU22, ASP23, ASN27. | **-6.06** | ASP23, VAL24, GLY25, SER26, ASN27, LYS28, GLY29, ILE31, ILE32, GLY33, LEU34, MET35. | **-5.07** | LEU17, VAL18, ALA21, GLU22, ASP23, VAL24, GLY25, SER26, ALA30 |
| F1S4-p | **-7.67** | GLU11, VAL12, HIS14, GLN15, VAL18, PH19, PHE20, GLU22, ASP23, VAL24, ASN27. | **-5.81** | PHE20, ALA21, GLU22, ASP23, VAL24, GLY25, SER26. | **-5.72** | HIS14, VAL18, PHE19, GLU22, ASP23. |
| F1S5-p | **-6.2** | ASP7, TYR10, GLU11, VAL12, HIS14, GLN15, VAL18, PHE19, GLU22, ASP23, ASN27 | **-5.44** | ALA21, GLU22, ASP23, VAL24, GLY25, ILE32, LEU34, VAL36 | **-4.17** | LEU17, VAL18, ALA21, GLU22, GLY25, SER26. |
| F1S6-p | **-6.25** | TYR10, GLU11, VAL12, HIS13, HIS14, GLN15, VAL18, PHE19, GLU22, ASP23, ASN27. | **-5.74** | ASP23, VAL24, GLY25, SER26, ASN27, LYS28, GLY29, ILE32, GLY33, LEU34, MET35. | **-4.78** | PHE20, ALA21, GLU22, GLY25, SER26, ASN27, LYS28, GLY29, ALA30, ILE31, ILE32, GLY33. |
| F1S7-p | **-5.87** | TYR10, GLU11, VAL12, HIS13, HIS14, GLN15, LYS16, VAL18, PHE19, PHE20, GLU22, ASP23, ASN27. | **-5.71** | ASP23, VAL24, GLY25, SER26, ASN27, LYS28, GLY29, ILE31, ILE32, GLY33, LEU34, MET35. | **-3.95** | LEU17, VAL18, ALA21, GLU22, ASP23, GLY25, SER26. |
| F1S8-p | **-4.4** | TYR10, GLU11, VAL12, HIS13, HIS14, GLN15, LYS16, VAL18, PHE19, PHE20, GLU22, ASP23, ASN27. | **-3.93** | ALA21, GLU22, ASP23, VAL24, GLY25, ILE32, LEU34. | **-3.98** | PHE20, ALA21, GLY25, SER26, LYS28, GLY29, ALA30, ILE31, ILE32, GLY33. |
| F2S1-p | **-4.95** | PHE19, PHE20, ASP23, VAL24, ASN27, LYS28, ILE31, ILE41. | **-4.37** | PHE19, PHE20, ALA21, GLU22, ASP23, VAL24, LEU34. | **-4.66** | PHE20, ALA21, GLU22, GLY25, SER26, ASN27, LYS28, GLY29, ALA30, ILE31, ILE32, GLY33. |
| F2S2-p | **-5.72** | YR10, GLU11, VAL12, HIS14, GLN15, VAL18, PHE19, PHE20, GLU22, ASP23, VAL24, ASN27 | **-4.6** | ALA21, GLU22, ASP23, GLY33, LEU34, MET35, VAL36. | **-4.25** | HIS14, GLN15, VAL19, GLU22, ASP23. |
| F2S3-p | **-5.72** | ASP7, TYR10, GLU11, VAL12, HIS14, GLN15, VAL18, PHE19, PHE20, GLU22, ASP23, ASN27. | **-4.92** | ASP23, VAL24, GLY25, LYS28, GLY29, ILE31, ILE32, GLY33, LEU34, MET35. | **-4.53** | GLU22, ASP23, VAL24, GLY25, SER26, ASN27, LYS28. |
| F2S4-p | **-7.74** | ASP7, TYR10, GLU11, VAL12, HIS14, GLN15, VAL18, PHE19, PHE20, GLU22, ASP23, VAL24, ASN27. | **-5.77** | PHE20, ALA21, GLU22, ASP23, VAL24, GLY25, SER26. | **-5.75** | HSD14, LEU17, VAL18, PHE19, GLU22, ASP23, VAL24, GLY25, SER26. |
| F2S5-p | **-5.98** | TYR10, GLU11, HIS14, GLN15, VAL18, PHE19, PHE20, GLU22, ASP23, ASN27. | **-5.22** | ALA21, GLU22, ASP23, VAL24, GLY25, ILE32, ILE34, VAL36. | **-4.1** | GLY9, HSD13, HSD14, LEU17, VAL18, ALA21, GLU22. |
| F2S6-p | **-5.92** | ASP7, TYR10, GLU11, VAL12, HIS14, GLN15, VAL18, PHE19, PHE20, GLU22, ASP23, AASN27. | **-4.91** | ASP23, VAL24, GLY25, SER26, LYS28, GLY29, ILE32, GLY33, LEU34, MET35. | **-4.72** | ALA17, PHE20, ALA21, GLU22, GLY25, SER26, LYS28, GLY29, ALA30, ILE31, ILE32. |
| F2S7-p | **-5.87** | TYR10, GLU11, VAL12, HIS13, HIS14, GLN15, LYS16, VAL18, PHE19, PHE20, GLU22, ASP23, ASN27. | **-5.09** | ASP23, VAL24, GLY25, ILE31, ILE32, GLY33, LEU34, MET35. | **-4.49** | PHE19, GLU22, ASP23, VAL24, GLY25, SER26, ASN27, LYS28. |
| F2S8-p | **-4.67** | GLU11, VAL12, HIS14, GLN15, VAL18, PHE19, PHE20, GLU22, ASP23. | **-3.86** | GLU22, ASP23, VAL24, GLY25, LEU34. | **-4.53** | PHE20, ALA21, GLU22, GLY25, SER26, ASN27, LYS28, GLY29, ALA30, ILE31, ILE32, GLY33 |
| F3S1-p | **-4.94** | PHE19, PHE20, ASP23, VAL24, ASN27, LYS28, ILE31, ILE41 | **-4.57** | ALA21, GLU22, ASP23, VAL24, GLY25, SER26, LYS28, GLY29, ILE32, GLY33, LEU34, MET35. | **-5.04** | ASP23, VAL24, GLY25, SER26, ASN27, LYS28. |
| F3S2-p | **-5.3** | ASP7, TYR10, GLU11, VAL12, HIS14, GLN15, VAL18, PHE19, PHE20, GLU22, ASP23. | **-4.75** | ALA21, GLU22, ASP23, ILE32, GLY33, LEU34, MET35, VAL36. | **-4.36** | HSD14, GLN15, VAL18, PHE19, GLU22, ASP23. |
| F3S3-p | **-5.72** | ASP7, TYR10, GLU11, VAL12, HIS14, GLN15, VAL18, PHE19, PHE20, GLU22, ASP23, ASN27. | **-4.93** | ASP23, VAL24, GLY25, LYS28, GLY29, ILE31, ILE32, GLY33, LEU34, MET35. | **-4.37** | GLU22, ASP23, VAL24, GLY25, SER26, ASN27, LYS28. |
| F3S4-p | **-7.58** | ASP7,, GLU11, VAL12, GLN15, VAL18, PHE19, PHE20, GLU22, ASP23, VAL24, ASN27. | **-5.48** | PHE20, ALA21, GLU22, ASP23, VAL24, GLY25, SER26. | **-5.79** | HSD14, VAL18, PHE19, GLU22, ASP23, VAL24, SER26. |
| F3S5-p | **-5.9** | TY10, GLU11, HIS14, GLN15, VAL18, PHE19, PHE20, GLU22, ASP23, ASN27. | **-4.6** | ALA21, GLU22, ASP23, VAL24, GLY25, ILE32, LEU34, VAL36. | **-4.1** | LEU17, VAL18, GLU22, GLY25, SER26. |
| F3S6-p | **-5.82** | GLU11, VAL12, HIS14, GLN15, VAL18, PHE19, PHE20, GLU22, ASP23, ASN27. | **-4.87** | ASP23, VAL24, GLY25, LYS28, GLY29, ILE31, ILE32, GLY33, LEU34, MET35, VAL36. | **-5.04** | LYS16, LEU17, VAL18, ALA21, GLU22, GLY25, LYS28, GLY29, ALA30, ILE31, ILE32. |
| F3S7-p | **-4.7** | TY10, GLU11, HIS14, GLN15, VAL18, PHE19, PHE20, GLU22, ASP23, ASN27. | **-4.66** | ALA21, GLU22, ASP23, VAL24, GLY33, LEU34, MET35, VAL36. | **-4.49** | LEU17, VAL18, PHE20, ALA21, GLU22, VAL24, GLY25, SER26, ASN27, LYS28, GLY29, ALA30, ILE31. |
| F3S8-p | **-4.14** | GLU11, VAL12, HIS14, GLN15, VAL18, PHE19, PHE20, GLU22, ASP23, ASN27. | **-3.81** | GLU22, ASP23, VAL24, GLY25, LEU34. | **-4.91** | PHE19, ASP23, VAL24, SER26, ASN27, LYS28. |
| F4S1-p | **-4.65** | PHE19, PHE20, ASP23, VAL24, ASN27, LYS28, ILE31, ILE41. | **-4.77** | ASP23, VAL24, GLY25, SER26, ASN27, LYS28, GLY29, ILE32, GLY33, LEU34, MET35. | **-4.67** | PHE19, ASP23, VAL24, GLY25, SER26, ASN27, LYS28. |
| F4S2-p | **-6.12** | ASP7, TYR10, GLU11, VAL12, HIS13, HIS14, GLN15, LYS16, VAL18, PHE19, PHE20, GLU22, ASP23, ASN27. | **-5.2** | GLU22, ASP23, VAL24, ILE32, GLY33, LEU34, MET35, VAL36. | **-4.55** | PHE19, ASP23, VAL24, SER26, ASN27 |
| F4S3-p | **-5.3** | ASP7, TYR10, GLU11, VAL12, HIS14, GLN15, VAL18, PH19, GLU22, ASP23, ASN27. | **-5.77** | ASP23, VAL24, GLY25, SER26, ASN27, LYS28, GLY29, ILE32, GLY33, LEU34, MET35. | **-4.74** | PHE19, GLU22, ASP23, VAL24, GLY25, SER26, ASN27. |
| F4S4-p | **-7.27** | TYR10, GLU11, VAL12, HIS13, HIS14, GLN15, VAL18, PHE19, PHE20, GLU22, ASP23, VAL24, ASN27. | **-5.54** | PHE20, ALA21, GLU22, ASP23, VAL24, GLY25, SER26. | **-5.9** | VAL18, GLU22, ASP23, VAL24, SER26. |
| F4S5-p | **-5.64** | TYR10, GLU11, VAL12, HIS14, GLN15, VAL18, PHE19, PHE20, GLU22, ASP23, ASN27. | **-5.08** | ASP23, VAL24, GLY25, SER26, ASN27, LYS28, GLY29, ILE32, GLY33, LEU34, MET35 | **-4.09** | HSD14, GLN15, VAL18, PH19, ASP23, VAL24. |
| F4S6-p | **-5.56** | ASP7, TYR10, GLU11, VAL12, HIS13, HIS14, GLN15, LYS16, VAL18, PHE19, PHE20, GLU22, ASP23, ASN27. | **-5.26** | ASP23, VAL24, GLY25, SER26, ASN27, LYS28, ILE31, ILE32, GLY33, LEU34, MET35. | **-4.45** | PHE19, GLU22, ASP23, VAL24, ASN27, GLY25. |
| F4S7-p | **-5.05** | TYR10, GLU11, VAL12, HIS14, GLN15, VAL18, PHE19, PHE20, GLU22, ASP23, ASN27. | **-5.17** | ASP23, VAL24, GLY25, SER26, ASN27, LYS28, GLY29, ILE32, GLY33, LEU34, MET35. VAL36 | **-4.3** | PHE19, GLU22, ASP23, VAL24, GLY25, SER26, ASN27, LYS28. |
| F4S8-p | **-4.09** | GLU11, VAL12, HIS14, GLN15, VAL18, PHE19, PHE20, GLU22, ASSP23, ASN27. | **-4.07** | ALA21, GLU22, ASP23, VAL24, GLY33, LEU34, MET35, VAL36. | **-4.28** | ASP23, VAL24, GLY25, SER26, ASN27, LYS28. |
| CUCURMIN | **-4.99** | GLN15, VAL18, PHE19, PHE20, GLU22, ASP23, VAL24, ASN27, LYS28 | **-5.57** | PHE19, ALA21, GLU22, ASP23, GLY33, LEU34, MET35, VAL36, GLY37, GLY38, VAL39 | **-4.6** | HSD13, HSD14, GLN15, VAL18, PHE19, ASP23, VAL24, SER26, ASN27, LYS28 |
| GALANTAMINE | **-5.85** | ASN27, ALA30, ILE31, ILE32, MET35, VAL39, VAL40, ILE41, ALA42 | **-5.79** | PHE20, ALA21, GLU22, ASP23, LEU34, MET35, VAL36, GLY37 | **-5.04** | PHE20, ALA21, GLU22, GLY25, SER26, ASN27, LYS28, GLY29, ALA30, ILE31, ILE32, |
| PI-IV | **-6.47** | PHE19, PHE20, ASP23, VAL24, ASN27, LYS28, ILE31, ILE41, ALA42 | **-7.4** | ASP23, VAL24, GLY25, SER26, ASN27, LYS28, GLY29, ILE32, GLY33, LEU34, MET35. VAL36 | **-5.85** | PHE19, GLU22, ASP23, VAL24, GLY25, SER26, ASN27, LYS28. |

**S2 Table.** Free energy values (kcal/mol) an amino acids o interact in a 5 Ǻ radius toward β-Secretase (BACE1) and human acetylcholinesterase (AChE)

| Compound | BACE1 | | AChE | |
| --- | --- | --- | --- | --- |
|  | **ΔG** | **AA** | **ΔG** | AA |
| F1S1-m | **-8.3** | LEU30, ASP32, SER35, TYR71, THR72, GLN73, GLY74, PHE108, ILE116, TYR198, ASP228, GLY230, THR231, ARG235, VAL332 |  |  |
| F1S2-m | **-8.13** | LEU30, ASP32, GLY34, SER35, PRO70, TYR71, THR72, GLN73, ILE118, TYR198, ILE226, ASP228, GLY230, THR291, ARG235, VAL332 |  |  |
| F1S3-m | **-7.98** | ASP32, GLY34, SER35, SER36, ASN37, VAL69, PRO70, TYR71, THR72, GLN73, ILE126, ALA127, ARG128, TYR198, ASP228, GLY230, THR231. | **-8.68** | TYR70, ASP72, TYR121, TRP279, SER286, ILE287, PHE288, ARG289, PHE290, PHE330, PHE331, TYR334, GLY335, ACH998 |
| F1S4-m | **-9.73** | ASP32, TH33, GLY34, SER35, SER36, ASN37, VAL69, PRO70, TYR71, THR72, GLN73, ILE126, ALA127, ARG128, TYR196, ASP228, SER229, GLY230, THR231, | **-9.58** | TYR70, VAL71, ASP72, GLU73, GLN74, GLY80, SER81, GLU82, TRP84, ASN85, TYR1212, SER122, PHE330, PHE331, TYR334. |
| F1S5-m | **-8.81** | SER10, GLY11, GLN12, GLY13, TYR14, LEU30, ASP32, GLY34, SER35, TYR71, THR72, GLN73, GLY74, LYS75, LYS107, PHE108, ILE110, TRP115, ILE118, ASP228, SER229, GLY230, THR231 THR232 |  |  |
| F1S6-m | **-8** | SER10, GLY11, GLN12, GLY13, LEU14, ASP32, GLY34, SER35, TYR71, THR72, GLN73, GLY74, LYS75, LYS107, PHE108, ILE110, TRP115, ILE118, ASP228, SER229, GLY230, THR231, THR232 |  |  |
| F1S7-m | **-7.79** | GLN12, GLY13, TYR14, LEU30, ASP32, GLY34, SER35, TYR71, THR72, GLN73, GLY74, LYS75, LYS107, PHE108, ILE110, TRP115, ILE118, ASP228. |  |  |
| F1S8-m | **-7.07** | ASP32, GLY34, SER35, THR72, TYR71, GLN73, TYR198, LYS224, ILE226, ASP228, THR231, ARG235, SER327, THR329, VAL332. |  |  |
| F2S1-m | **-8.14** | ASP32, GLY34, SER35, PRO70, TYR71, THR72, GLN73, ILE118, TYR198, LYS226, ILE226, ASP228, GLY230, THR231, THR329 |  |  |
| F2S2-m | **-8.72** | LEU30, ASP32, GLY34, SER 35, PRO70, TYR71, THR72, GLN73, ILE118, TYR198, ILE226, ASP228, GLY230, THR231, ARG235, VAL332. |  |  |
| F2S3-m | **-8.39** | GLN12, GLY13, LEU30, ASP32, GLY34, SER35, ASP62, ARG64, TYR71, THR72, GLN73, GLY74, LYS75, LYS107, PHE108, ILE110, TRP115, ILE118, TYR298, GLY230, THR231. | **-8.6** | GLN69, TYR70, VAL71, ASP72, SER81, TRP84, ASN85, PRO86, TYR121, SER122, GLY123, SER124, TRP279, PHE288, PHE330, PHE331, TYR334. |
| F2S4-m | **-9.5** | LEU30, ASP32, THR33, GLY34, SER35, TYR71, THR72, GLN73, GLY74, PHE108, ILE110, TRP115, ASP228, SER229, ARG235. | **-9.69** | ASP74, GLY82, THR83, TRP86, GLY120, GLY121, GLY122, TYR124, SER125, TYR133, GLU202, SER203, ALA204, TYR337, TYR341, TRP439, PRO446, HIS447, GLY448, TYR449, ILE451. |
| F2S5-m | **-8.82** | SER10, GLY11, GLN12, GLY13, TYR714, LEU30, ASP32, GLY34, SER35, TYR71, THR72, GLN73, GLY74, LYS75, ASP106, LYS107, PHE108, ILE110, TRP115, ILE118, ASP228, SER229, GLY230, THR231 THR232, ALA275. |  |  |
| F2S6-m | **-8.89** | ASP32, GLY34, SER35, SER36, ASN37, VAL69, PRO70, TYR71, THR72, GLN73, ILE118, ILE126, ALA127, ARG128, ASP228, GLY230, THR231 |  |  |
| F2S7-m | **-8.65** | ASP32, GLY34, SER35, SER36, ASN37, VAL69, PRO70, TYR71, THR72, GLN73, TYR98, ILE118, ILE126, ALA127, ARG128, ASP228, GLY230, THR231. |  |  |
| F2S8-m | **-7.68** | ASP32, GLY34, SER35, SER36, PRO70, TYR71, THR72, GLN73, ILE126, ARG128, TYR198, ASP228, GLY230- |  |  |
| F3S1-m | **-7.96** | ASP32, GLY34, SER35, TYR171, THR72, ARG235, SER327, THR329, VAL332. |  |  |
| F3S2-m | **-8.2** | LEU30, ASP32, GLY34, TYR71, THR72, GLN73, GLY74, PHE108, ILE118, TYR198, ILE226, ASP228, GLY230, THR232, ARG235, THR329, VAL332 |  |  |
| F3S3-m | **-8.12** | GLN12, GLY13, LEU30, ASP32, GLY34, SER35, TYR71, THR72, GLN73, GLY74, LYS75, LYS107, PHE108, ILE110, TRP115, ILE118, ASP228, GLY230. | **-8.67** | TYR70, ASP72, TYR121, SER122, TRP279, LEU282, SER286, ILE287, PHE288, ASRG289, PHE290, PHE330, PHE331, TYR334, GLY335. |
| F3S4-m | **-9.89** | ASP32, THR33, GLY34, SER35, SER36, ASN37, VAL69, PRO70, TYR71, THR72, GLN73, ILE126, ALA127, ARG128, ASP228, SER 229 GLY230, THR231 | **-10.06** | ASP74, GLY82, THR83, TRP86, GLY120, GLY121, GLY122, TYR124, TYR133, GLU202, SER203, ALA204, TYR337, TYR341, TRP439, PRO446, HIS447, GLY448, TYR449, ILE451. |
| F3S5-m | **-8.95** | SER10, GLY11, GLN12, GLY13, TYR14, LEU30, ASP32, GLY34, SER35, TYR71, THR72, GLN73, GLY74, LYS75, LYS107, PHE108, ILE110, TRP115, ILE118, ASP228, SER229, GLY230, THR231, THR232 |  |  |
| F3S6-m | **-8.24** | SER10, GLN12, GLY13, LEU30, ASP32, GLY34, SER35, TYR71, THR72, GLN73, GLY74, LYS75, LYS107, PHE108, ILE110, TRP115, ILE118, ASP228, SER229, GLY230, THR231, THR232. |  |  |
| F3S7-m | **-8.41** | ASP32, GLY34, SER35, SER36, ASN37, VAL69, PRO70, TYR71, THR72, GLN73, ILE1126, ALA127, ARG128, GLY230, THR231. |  |  |
| F3S8-m | **-6.76** | ASP32, GLY34, SER35, TYR71, THR72, GLN73, TYR198, LYS224, ILE226, ASP228, GLY230, THR231, ARG235, SER327, THR329. |  |  |
| F4S1-m | **-7.74** | ASP32, GLY34, SER35, SER36, ASN37, VAL69, PRO70, TYR71, THR72, GLN73, ILE118, ILE126, ALA127, ARG128, ASP228, GLY230, THR231 |  |  |
| F4S2-m | **-7.77** | LEU30, ASP32, GLY34, SER35, TYR71, THR72, GLN73, GLY74, LYS75, TRP76, PHE108, PHE109, ILE110, TRP115, ILE118, ASP228, THR231, THR232. |  |  |
| F4S3-m | **-8.19** | GLN12, GLY13, LEU30, ASP32, GLY34, SER35, TYR71, THR72, GLN73, GLY74, LYS75, ASP106, LYS107, PHE108, PHE109, ILE110, TRP115, ILE118, TYR198, ASP228, GLY230, THR231. | **-7.97** | ASP72, SER81, TRP84, ASN85, TYR121, SER122, TRP279, SER286, ILE287, PHE288, ARG289, PHE290, PHE330, PHE331, TYR334, GLY335. |
| F4S4-m | **-8.97** | ASP32, THR33, GLY34, SER35, SER36, ASN37, VAL69, PRO70, TYR71, THR72, GLN73, ILE126, ALA127, ARG128, TYR198, ASP228, SER229, GLY230, THR231 | **-9.58** | ASP72, GLY80, SER81, GLU82, MET83, TRP84, ASN85, GLY118, GLY119, TYR121, SER122, TRP279, ILE287, SER286, PHE288, ARG289, PHE290, PHE330, PHE331, TYR334, GLY335TRP432. |
| F4S5-m | **-8.75** | SER10, GLY11, GLN12, GLY13, TYR14, LEU30, ASP32, GLY34, SER35, TYR71, THR72, GLN73, GLY74, LYS75, LYS107, PHE108, ILE110, TRP115, ILE118, ASP228, SER229, GLY230, THR231, THR293, ALA335 |  |  |
| F4S6-m | **-8.22** | ASP32, GLY34, SER35, SER36, ASN37, VAL69, PRO70, TYR71, THR72, GLN73, ILE118, ILE126, ARG128, TYR198, ASP228, GY230, THR31 |  |  |
| F4S7-m | **-8.3** | AS32, GLY34, SER35, SER36, VAL69, PRO70, TYR71, THR72, GLN73, ILE126, ALA127, ARG128, TYR198, ASP228, GLY230, THR231. |  |  |
| F4S8-m | **-7.42** | LEU30, ASP32, GLY34, SER35, ARG64, TYR71, THR72, GLN73, LYS75, LYS107, PHE108, ILE110, TRP115, ILE118, TYR198, ASP228, GLY230, THR231 |  |  |
| F1S1-p | **-8.24** | LEU30, ASP32, GLY34, SER35, TYR71, THR72, GLN73, GLY74, PHE108, ILE118, TYR198, ASP228, GLY230, THR231, ARG235, SER327, THR329, VAL332. |  |  |
| F1S2-p | **-8.86** | LEU30, ASP32, GLY34, SER35, TYR71, THR72, GLN73, GLY74, LYS75, LYS107, PHE108, PHE109, ILE110, TRP115, ILE179, ASP228, GY230, THR231. |  |  |
| F1S3-p | **-8.04** | LEU30, ASP32, GLY34, SER35, TYR71, THR72, GLN73, GLY74, PHE108, TRP115, ILE118, TYR2198, ILE226, ASP228, GLY230, THR231, ARG235, THR329, VAL332. | **-8.53** | TRP86, GLY120, GLY121, GLY122, TYR124, SER125, TYR133, GLU202, SER203, ALA204, TRP286, PHE295, PHE297, TYR337, PHE338, HIS447, GLY448, TYR449, ILE451. |
| F1S4-p | **-9.81** | LEU30, ASP32, THR33, GLY34, SER35, TYR71, THR72, GLN73, GLY74, LYS107, PHE108, ILE110, TRP115, ILE118, ASP228, SER229, GLY230, THR231. | **-9.21** | ASP74, GLY82, THR83, GLU84, TRP86, ASN87, GLY120, GLY121, GLY122, TYR124, SER125, TYR133, GLU202, SER203, ALA204, TYR337, TYR341, TRP439, HIS447, GLY448, TYR449, ILE451. |
| F1S5-p | **-8.16** | LEU30, ASP32, GLY34, SER35, TYR71, THR72, GLN73, GLY74, PHE108, ILE118, TYR198, ILE226, ASP228, GLY230, THR231, ARG235, SER327, THR329, VAL332. |  |  |
| F1S6-p | **-8.72** | LEU30, ASP32, GLY34, SER35, TYR71, THR72, GLN73, GLY74, PHE108, ILE118, TYR198, LYS224, ILE226, ASP228, GLY230, THR231, ARG235, SER327, THR329, VAL332. |  |  |
| F1S7-p | **-8.32** | ASP32, GLY34, SER35, SER36, ASN37, VAL69, PRO70, TYR71, THR72, GLN73, ILE126, ALA127, ARG128, TYR198, ILE226, ASP228, THR231. |  |  |
| F1S8-p | **-7.5** | ASP32, GLY34, SER35, TYR71, THR72, GLN73, ILE179, TYR198, LYS224, ILE226, ASP228, GLY230, THR231, ARG235, THR329, VAL332. |  |  |
| F2S1-p | **-7.78** | ASP32, GLY34, SER35, PRO70, TYR71, THR72, GLN73, ILE108, TYR198, LYS224, ILE226, ASP228, GLY230, THR231, SER327, THR329, GLY330, VAL332, |  |  |
| F2S2-p | **-8.25** | LEU30, ASP32, GLY34, SER35, TYR71, THR72, GLN73, GLY74, LYS75, ASP106, LYS107, PHE108, PHE109, ILE110, TRP115, ILE118, TYR198, ASP228, GLY230, THR231. |  |  |
| F2S3-p | **-7.31** | LEU30, ASP32, GLY34, SER35, TYR71, THR72, GLN73, GLY74, LYS107, PHE108, ILE110, TRP115, ILE118, ILE226, ASP228, GLU230, THR231, ARG235, VAL332. | **-8.26** | ASP74, GLY82, THR83, GLU84, TRP86, ASN87, GLY120, GLY121, GLY122, TYR124, TYR133, GLU202, SER203, ALA204, TYR337, TYR341, TRP439, HIS447, PRO446, GLU448, TYR449, IE451. |
| F2S4-p | **-10** | ASP32, THR33, GLY34, SER335, SER36, ANS37, VAL69, PRO70, TYR71, THR72, GLN73, ILE126, ALA127, ARG128, ASP228, SER229, GLY230, THR231. | **-9.96** | ASP74, THR83, GLU84, TRP86, ASN87, GLY121, TYR124, SER125, GLU202, SER203, ALA204, TYR337, TYR341, TRP439, PRO446, HIS447, GLY448, TYR449, ILE451. |
| F2S5-p | **-8.45** | ASP32, GLY34, SER35, SER36, ASN37, VAL38, PRO70, TYR71, THR72, GLN73, ILE126, ALA127, ARG128, TYR198, ILE226, ASP228, GLY230, THR231. |  |  |
| F2S6-p | **-8.29** | LEU30, ASP32, SER35, TYR71, THR72, GLN73, GLY74, ILE118, TYR198, LYS224, ILE226, ASP228, GLY230, THR231, ARG235, SER327, VAL332. |  |  |
| F2S7-p | **-8.3** | ASP32, GLY34, SER35, SER36, ASN37, VAL69, PRO70, TYR71, THR72, GLN73, ILE126, ALA127, ARG128, TYR198, ILE226, ASP228, GLY230, THR231. |  |  |
| F2S8-p | **-7.77** | LEU30, ASP32, GLY34, SER35, TYR71, THR72, GLN73, GLY74, LYS75, ASP106, LYS107, PHE108, ILE110, TRP115, ILE118, ASP228, GLY230, THR231. |  |  |
| F3S1-p | **-8.12** | LEU30, ASP32, GLY34, SER35, TYR71, THR72, GLN73, GLY74, ILE118, TYR198, ASP228, GLY230, THR231, ARG235, THR329, VAL332. |  |  |
| F3S2-p | **-8.28** | LEU30, ASP32, GLY34, SER35, TYR71, THR72, GLN73, GLY74, LYS75, ASP106, LYS107, PHE108, PHE109, TRP115, ILE118, TYR198, ASP228, GLY230, THR231. |  |  |
| F3S3-p | **-7.59** | ASP32, GLY34, SER35, PRO70, TYR71, THR72, GLN73, GLY74, ILE118, TYR198, ILE226, THR231, ARG235, THR329, VAL332. | **-7.9** | TRP86, GLY120, GLY121, GLY122, TYR124, SER125, GLU202, SER203, ALA204, TRP236, PHE297, TYR337, PHE338, TYR341, TRP439, HIS447, GLY448, TYR449. |
| F3S4-p | **-9.76** | LEU30, ASP32, THR33, GLY34, SER35, TYR71, THR72, GLN73, GLY74, LYS107, PHE108, ILE110, TRP115, ILE118, ASP228, SER229, GLY230, THR231. | **-10.16** | ASP74, GLY82, THR83, GLU84, TRP86, ASN87, GLY120, GLY121, GLY122, TYR124, TYR133, GLU202, SER203, ALA204, TYR337, TYR341, TRP439, PRO446, HIS447, GLY448, TYR449, ILE451. |
| F3S5-p | **-8.48** | ASP32, GLY34, SER35, SER36, ASN37, VAL169, PRO170, TYR71, THR72, GLN73, ILE126, ALA127, ARG128, TYR198, ILE226, ASP228, GLY230, THR231. |  |  |
| F3S6-p | **-7.99** | LEU30, ASP32, GLY34, SER35, TYR71, THR72, GLN73, GLY74, LYS107, PHE108, PHE109, ILE110, TRP115, ILE118, TYR198, ILE226, ASP228, GLY230, THR231, ARG235. |  |  |
| F3S7-p | **-8.42** | ASP32, GLY34, SER35, SER36, ASN37, VAL69, PRO70, TYR71, THR72, GLN73, ILE126, ALA127, ARG128, TYR198, ILE226, ASP228, GLY230, THR231. |  |  |
| F3S8-p | **-7.59** | LEU30, ASP32, GLY34, SER35, TYR71, THR172, GLN73, GLY74, PHE108, ILE118, TYR198, ILE226, ASP228, GLY230, THR231, SER327, THR329, VAL332. |  |  |
| F4S1-p | **-8.36** | ASP32, GLY34, SER35, PRO70, TYR71, THR72, GLN73, ILE118, TYR198, TYR199, VAL191 ILE192, ASP223, LYS224, SER225, ILE226, VAL227, ASP228, SER229, GLY230, THR231, GLY330. VAL393. |  |  |
| F4S2-p | **-8.99** | GLN12, LEU30, ASP32, GLY34, SER35, TYR71, THR72, GLN73, GLY74, LYS107, PHE108, PHE109, ILE110, TRP115, ILE118, TYR198, GLY230, THR231. |  |  |
| F4S3-p | **-7.98** | LEU30, ASP32, GLY34, SER35, TYR71, THR72, GLN73, GLY74, LYS75, LYS107, PHE108, PHE109, TRP115, ILE118, TYR198 ILE226, ASP228, GLY230, THR231. | **-8.48** | GLY120, GLY121, GLY122, TYR124, SER125, SER203, ALA204, TRP286, LEU289, ARG296, SER293, AL294, ARG296, PHE297, TYR337, PHE338, TYR341, HIS447. |
| F4S4-p | **-9.45** | SER10, GLY11, GLN12, GLY13, LEU30, ASP32, THR33, GLY34, SER35, TYR71, THR133, GLN73, GLY74, PHE108, ILE110, TRP115, ILE118, ASP228, SER229, GLY230, THR231. | **-8.84** | TRP86, GLY120, GLY121, GLY122, TYR124, SER125, TYR133, GLU202, SER203, ALA204, TRP236, TRP286, PHE295, PHE297, TYR337, PHE338, HIS447, GLY448, ILE451. |
| F4S5-p | **-8.58** | LEU30, ASP32, GLY34, SER35, TYR71, THR72, GLN73, GLY74, LYS107, PHE108, ILE110, TRP115, ILE118, TYR198, ILE126, ASP228, GLY230, THR231. |  |  |
| F4S6-p | **-8.45** | LEU30, ASP32, GLY34, SER35, TYR71, THR72, GLN73, GLY74, LYS107, PHE108, PHE109, ILE110, TRP115, ILE118, TYR198, ILE226, ASP228, GLY230, THR231, ARG235. |  |  |
| F4S7-p | **-8.33** | LEU30, ASP32, GLY34, SER35, TYR71, THR72, GLN73, GLY74, LYS75, ASP106, LYS107, PHE108, PHE109, ILE110, TRP115, TYR198, ILE226, ASP228, GLY230. |  |  |
| F4S8-p | **-7.05** | LEU30, ASP32, GLY34, SER35, TYR71, THR72, GLN73, GLY74, LYS75, ASP106, LYS107, PHE108, ILE110, TRP115, ILE118, ASP228, GLY230, THR231. |  |  |
| CURCUMINE | **-9.46** | GLN12, GLY13, LEU30, ASP32, GLY34, SER35, SER36, ASN37, VAL69, PRO70, TYR71, THR72, GLN73, GLY74, PHE108, PHE109, PHE110, TRP115, ILE118, ILE126, ALA127, ARG128, ASP228, GLY230, THR231. |  |  |
| GALANTAMINE | **-7.66** | SER10, GLY11, GLN12, GLY13, TYR14, LEU30, GLN73, LYS107, PHE108, ILE110, TRP115, SER229, GLY230, THR231, THR232, ALA335 | **-9.07** | GLY82, TRP86, TRP117, GLY120, GLY121, GLY122, TYR124, SER125, TYR133, GLU202, SER203, ALA204, PHE297, TYR337, PHE338, TYR341, TRP439, HIS447, GLY448, TYR449, ILE451. |
| PI-IV | **-11.33** | SER71, GLY72, GLN72, GLY74, TYR75, LEU91, ASP93, GLY95, SER96, SER97, VAL130, PRO131, TYR132, THR133, GLN134, GLY135, LYS168, PHE169, PHE170, ILE171, TRP176, ILE179, ILE187, TYR259, LYS285, ILE287, SASP289, SER290, GLY291, THR292, THR293, ASN294, ARG296, SER388, THR390, VAL393. |  |  |


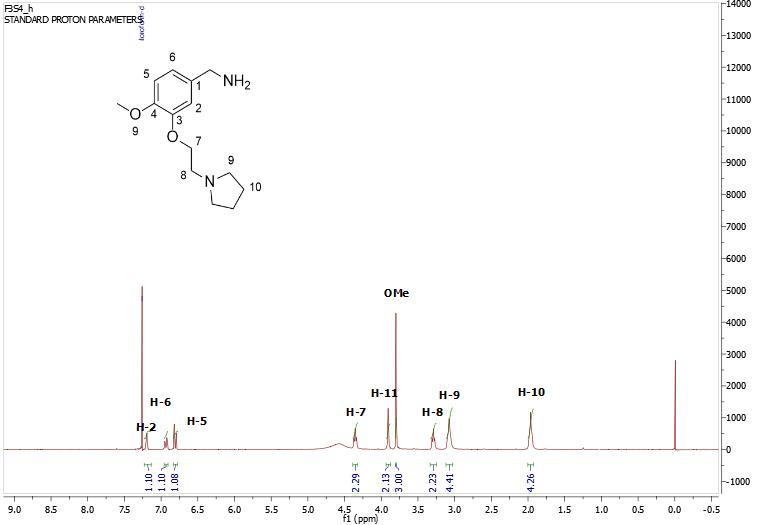


**S1 Fig.** ^1^H NMR spectrum of (4-methoxy-3- (2-(pyrrolidin-1-yl) ethoxy) phenyl) methanamine (**F3S4-m**) 300 MHz, CDCl_3_.


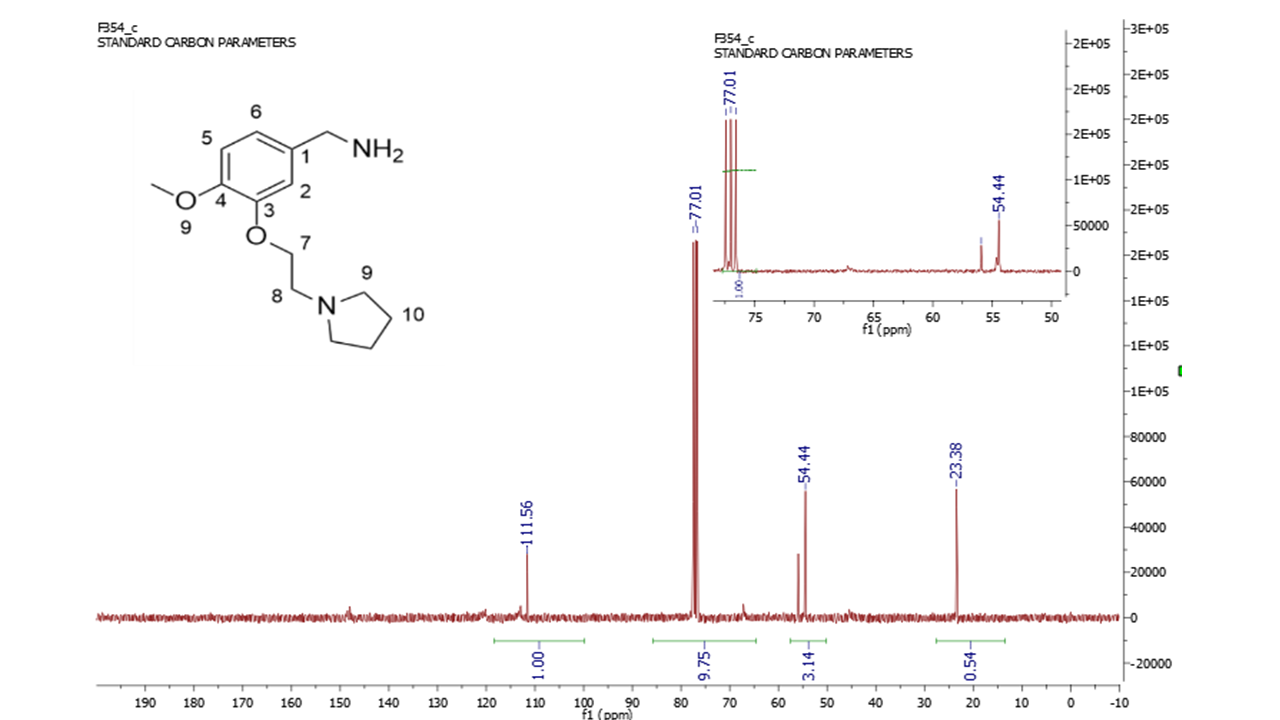


**S2 Fig.** ^13^C NMR spectrum of (4-methoxy-3- (2-(pyrrolidin-1-yl) ethoxy) phenyl) methanamine (**F3S4-m**) 75.45 MHz, CDCl_3_.


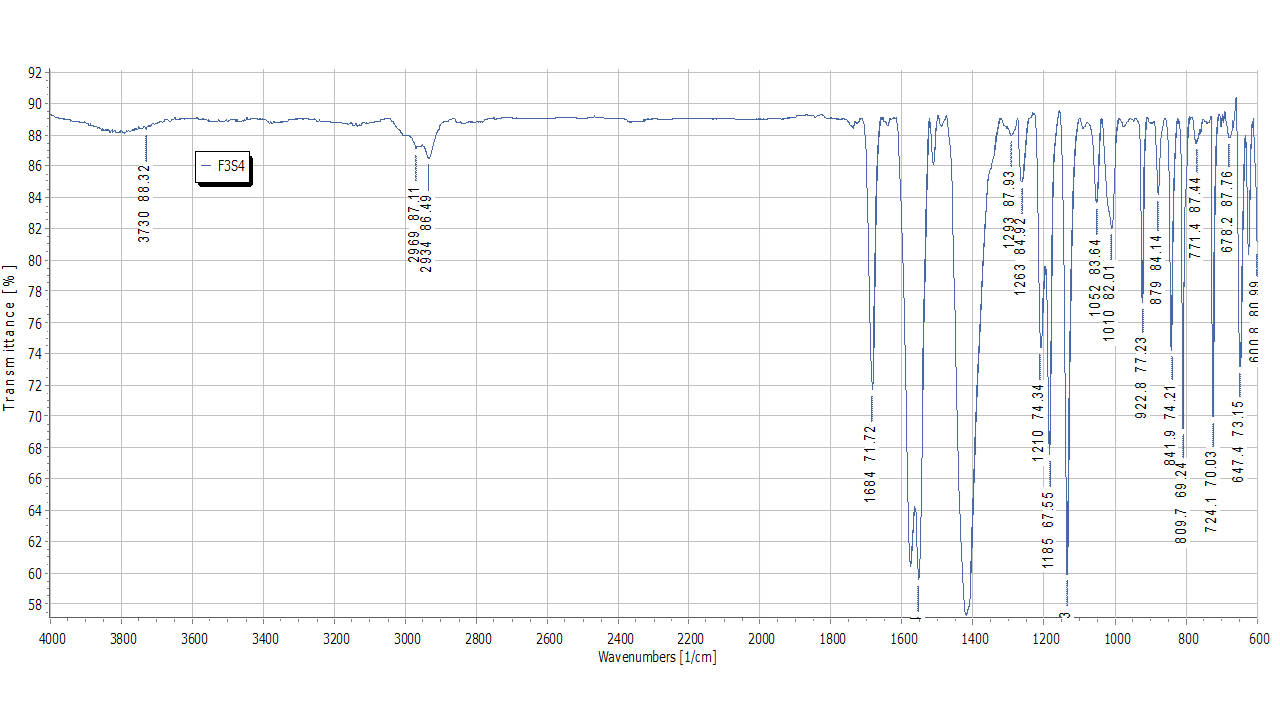


**S3 Fig**. IR spectra of (4-methoxy-3- (2-(pyrrolidin-1-yl) ethoxy) phenyl) methanamine (**F3S4-m**).


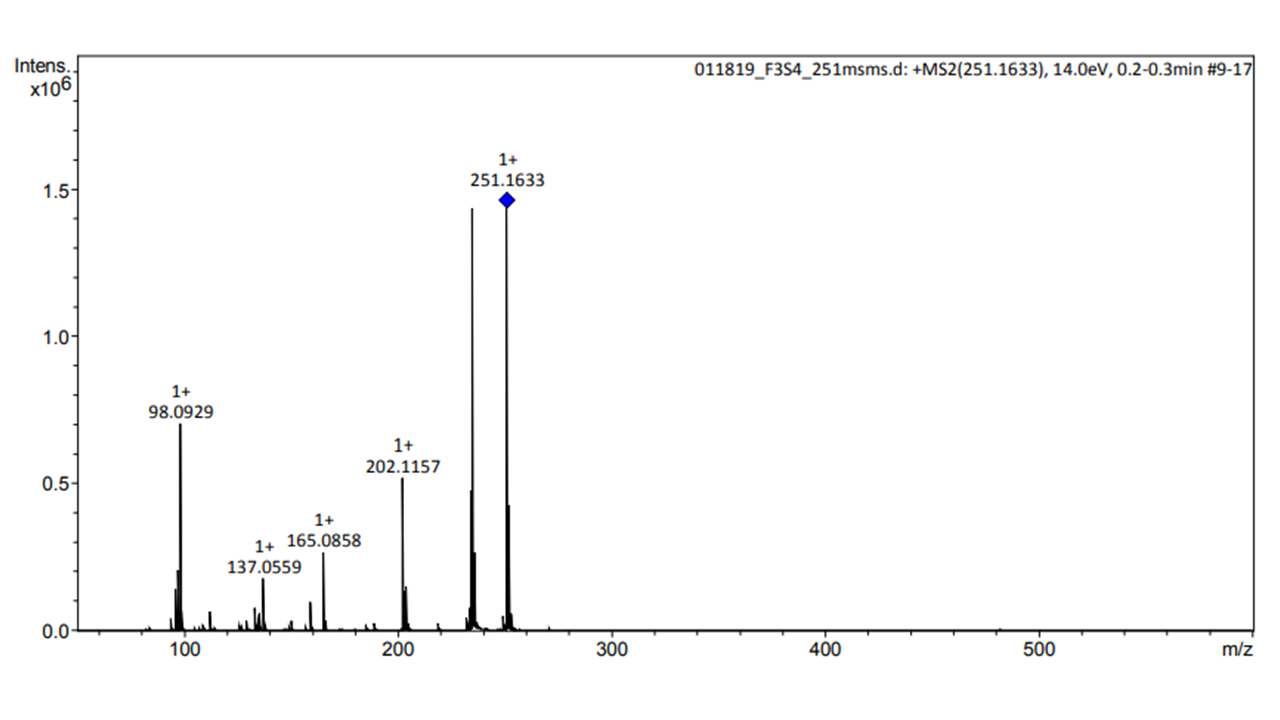


**S4 Fig.** Electron ionization mass spectrum using mass spectrometry to determine m/z of (4-methoxy-3- (2-(pyrrolidin-1-yl) ethoxy) phenyl) methanamine (**F3S4-m**).


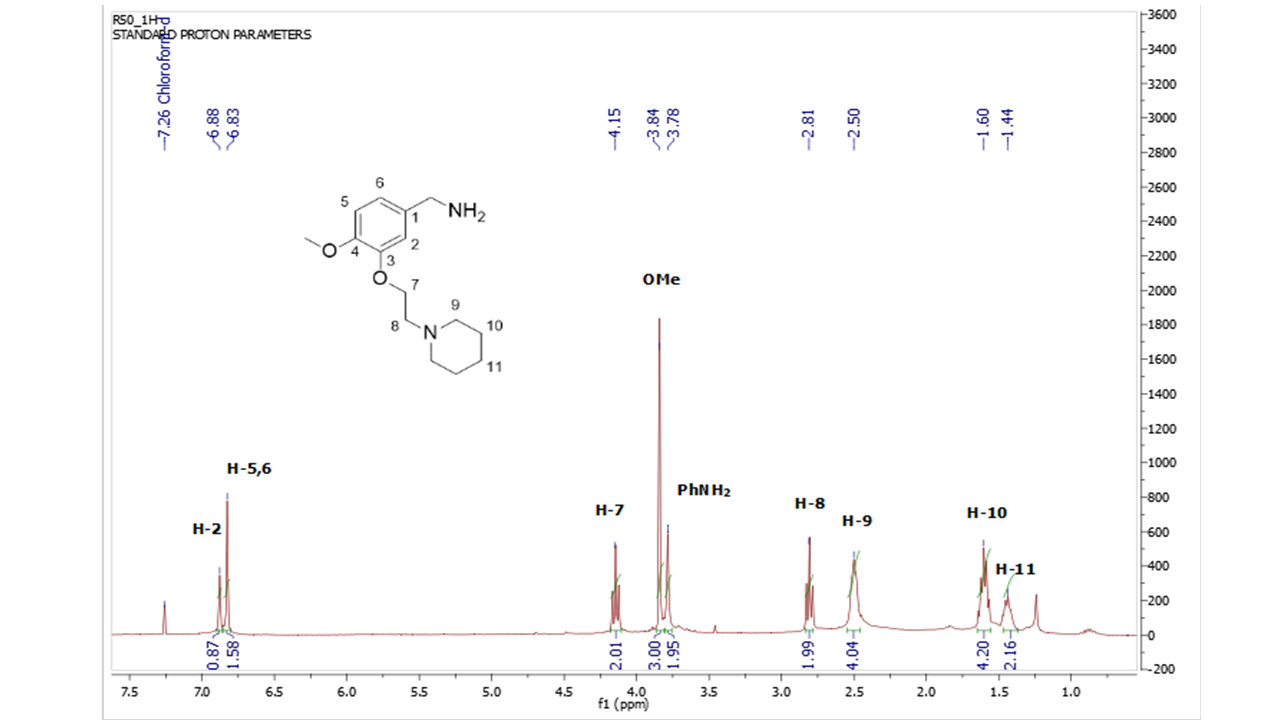


**S5 Fig.** ^1^H NMR spectrum of (4-methoxy-3-(2-(piperidin-1-yl) ethoxy) phenyl) methanamine (**F2S4-m**) 300 MHz, CDCl_3_.


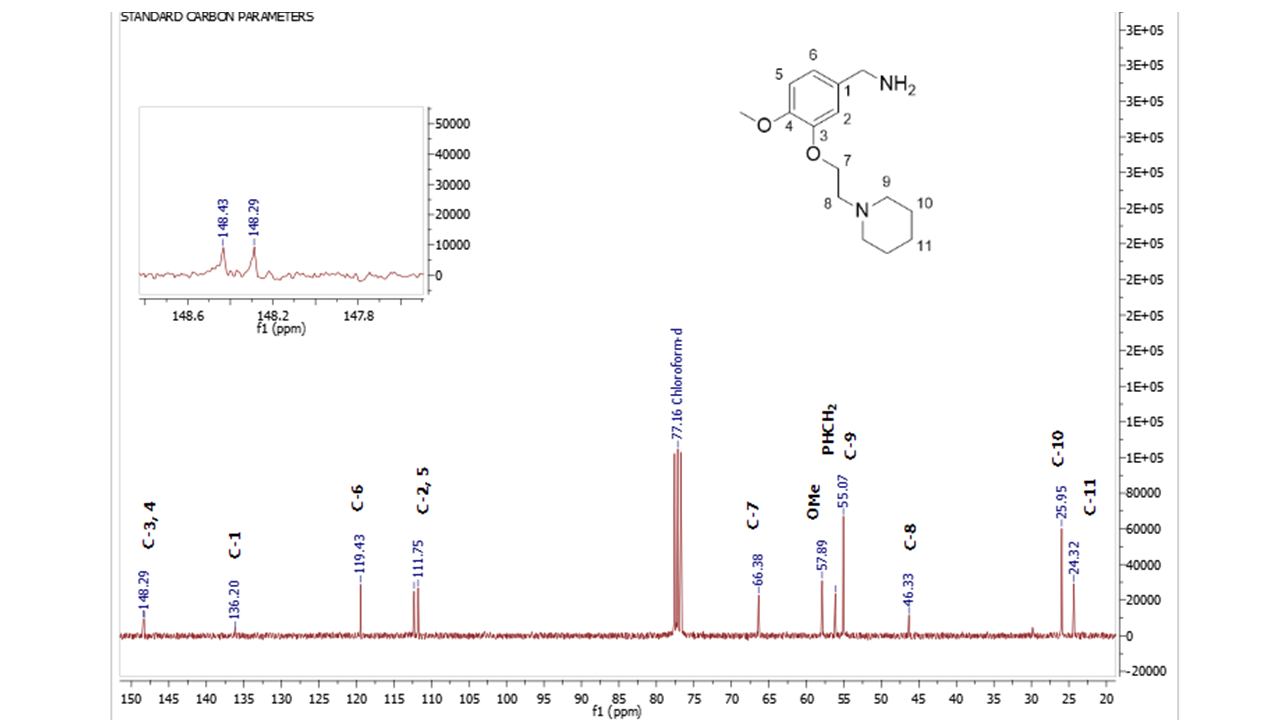


**S6 Fig**. ^13^C NMR spectrum of (4-methoxy-3-(2-(piperidin-1-yl) ethoxy) phenyl) methanamine (**F2S4-m**) 75.45 MHz, CDCl_3_.


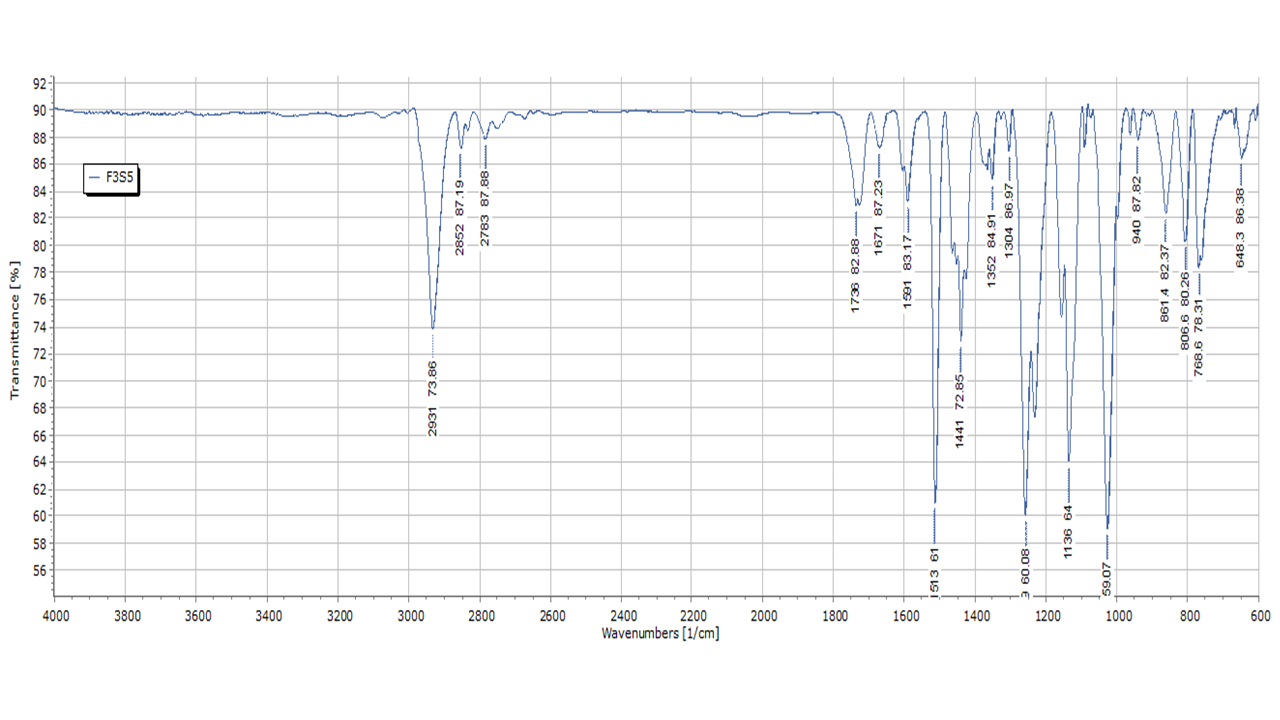


**S7 Fig**. Infrared (IR) spectra of (4-methoxy-3-(2-(piperidin-1-yl) ethoxy) phenyl) methanamine (**F2S4-m**).


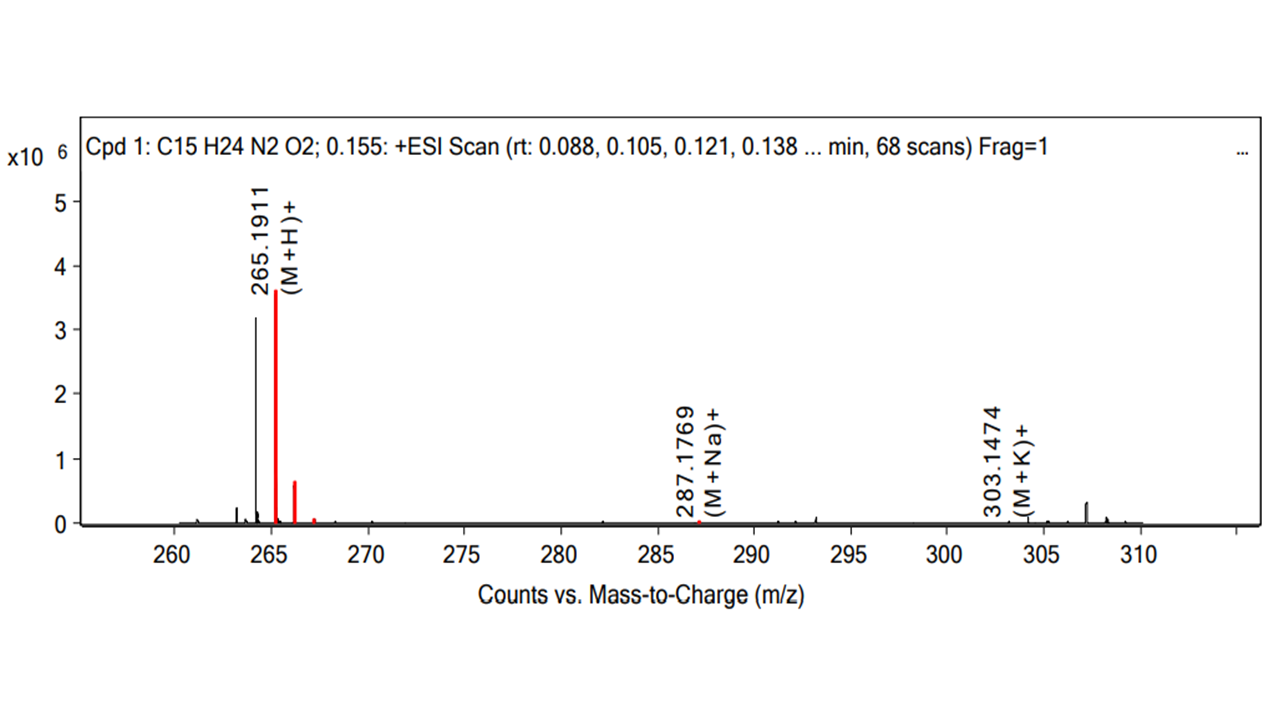


**S8 Fig**. Electron ionization mass spectrum using mass spectrometry to determine m/z of (4-methoxy-3-(2-(piperidin-1-yl) ethoxy) phenyl) methanamine (**F2S4-m**).


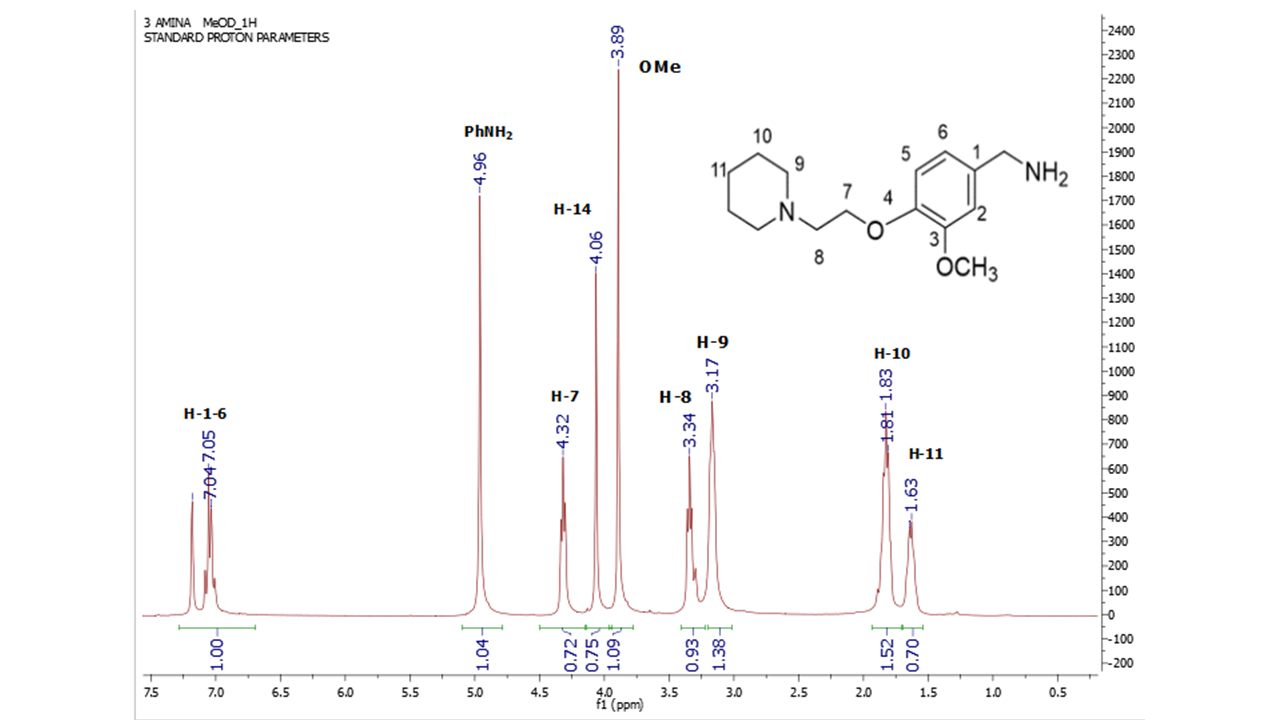


**S9 Fig**. ^1^H NMR spectrum of (3-methoxy-4-(2-(piperidin-1-yl) ethoxy) phenyl) methanamine (**F2S4-p**) 300 MHz, CDCl_3_.


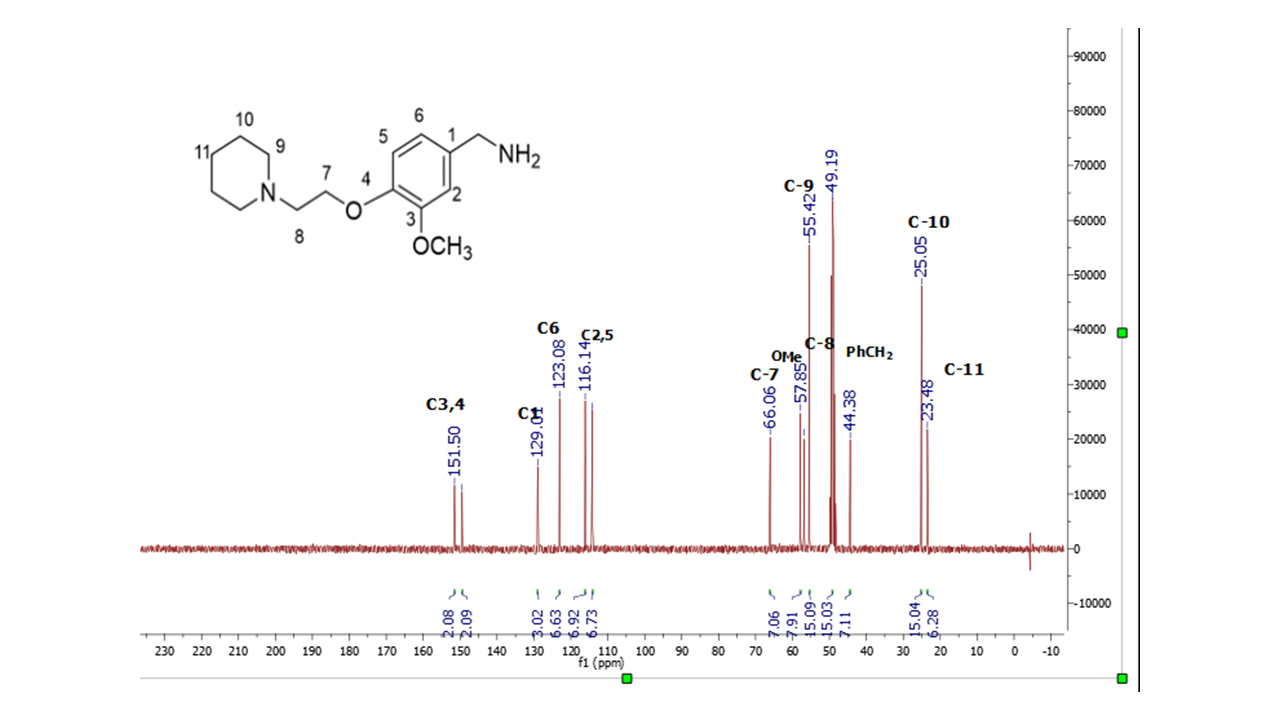


**S10 Fig.** ^13^C NMR spectrum of (3-methoxy-4-(2-(piperidin-1-yl) ethoxy) phenyl) methanamine (**F2S4-p**) 300 MHz, CDCl_3_.

.


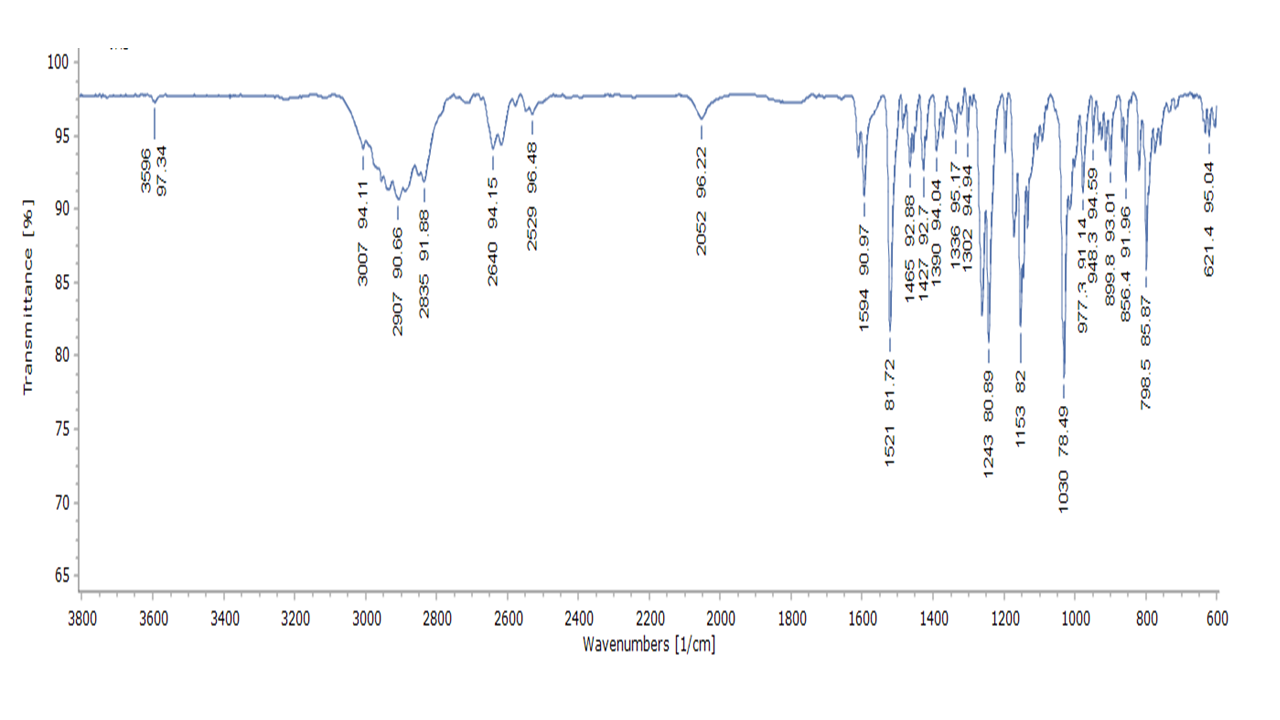


**S11 Fig**. Infrared (IR) spectra of (3-methoxy-4-(2-(piperidin-1-yl) ethoxy) phenyl) methanamine **(F2S4-p**).


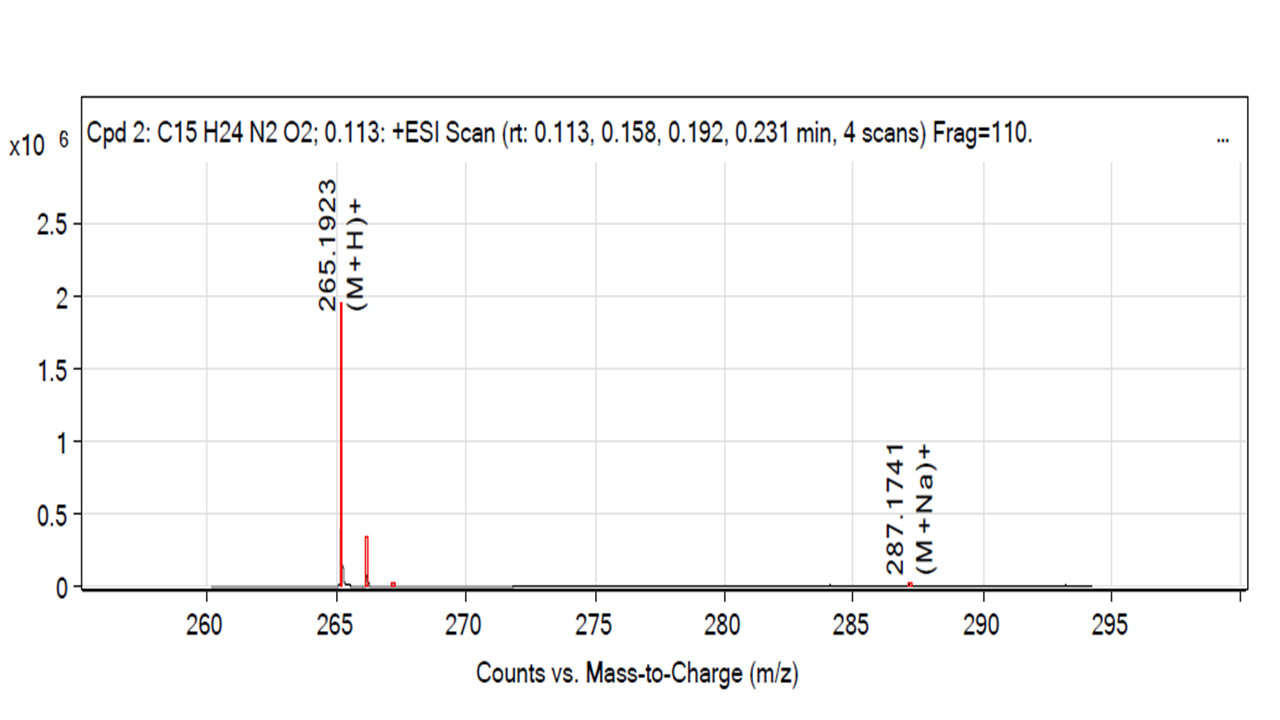


**S12 Fig.** Electron ionization mass spectrum using mass spectrometry to determine m/z of (3-methoxy-4-(2-(piperidin-1-yl) ethoxy) phenyl) methanamine (**F2S4-p**).

**S13 Fig.** Cell viability of astrocytes cell cultures in the presence of Cadmium chloride (0.01, 0.1, 1, 10, 100 uM) as control for cytotoxicity effect.
